# Discovery of Pyranoviolin A and Its Biosynthetic Gene Cluster in *Aspergillus violaceofuscus*

**DOI:** 10.3389/fmicb.2020.562063

**Published:** 2020-10-07

**Authors:** Xingxing Wei, Lin Chen, Jian-Wei Tang, Yudai Matsuda

**Affiliations:** ^1^Department of Chemistry, City University of Hong Kong, Kowloon, Hong Kong, China; ^2^Department of Ocean Science and Division of Life Science, Hong Kong University of Science and Technology, Clear Water Bay, Hong Kong, China; ^3^City University of Hong Kong Shenzhen Research Institute, Shenzhen, China

**Keywords:** *Aspergillus violaceofuscus*, natural products, biosynthesis, fungal secondary metabolites, polyketide-non-ribosomal peptides

## Abstract

A new polyketide-non-ribosomal peptide hybrid molecule, pyranoviolin A (**1**), was discovered from the genome-sequenced fungus *Aspergillus violaceofuscus* CBS 115571 and was characterized to be the first pyranonigrin analog harboring the C-3 methoxy group. Examination of the genome sequence of the fungus identified a putative biosynthetic gene cluster of **1**, which was designated as the *pyv* cluster. The gene deletion experiment of the polyketide synthase (PKS)-non-ribosomal peptide synthetase (NRPS) hybrid gene in the cluster confirmed the involvement of the *pyv* cluster in the pyranoviolin A biosynthesis. Finally, a plausible biosynthetic route leading to **1** has been proposed based on the bioinformatic analysis. Our study indicates that metabolite analysis of genome-sequenced microorganisms whose metabolites have been largely unexplored facilitates the discovery of new secondary metabolites along with their biosynthetic gene clusters.

## Introduction

Filamentous fungi have been rich sources for naturally occurring organic compounds and thus provided many pharmaceutical drugs, as exemplified by penicillins, cyclosporin, and lovastatin. However, since a large number of natural products have been isolated and characterized over the past century, it is becoming challenging to obtain novel natural products only with traditional methodologies. As more and more microbial genomes have been sequenced, many researchers have been seeking to obtain novel metabolites by activating silent and unexploited biosynthetic gene clusters. This approach, generally described as “genome mining,” has been widely utilized, leading to the discovery of many new natural products (Brakhage and Schroeckh, 2011; Rutledge and Challis, 2015; Zarins-Tutt et al., 2016; Yan et al., 2020). To activate a specific gene cluster, several different strategies could be used, including the overexpression of the pathway-specific transcriptional factor and heterologous expression of the gene cluster. Nevertheless, these strategies do not always work, probably due to the presence of “dead,” rather than “silent,” gene clusters (Montiel et al., 2015).

Meanwhile, we can now access the genome sequences of many microbial organisms whose metabolites have not been, or rarely, investigated. Thus, it is suggested that the metabolomic analysis of these microbial organisms leads to the rapid identification of new natural products. Additionally, because of the known genome sequences, the biosynthetic gene cluster of the new metabolite could be readily identified, and the biosynthetic study can be performed quickly after the isolation of the compound (Cacho et al., 2015). Importantly, the rapid linking of secondary metabolites to their biosynthetic gene clusters facilitates biosynthetic engineering of important compounds and mining of other related natural products.

In this study, to examine this concept, we investigated the metabolites produced by the fungus *Aspergillus violaceofuscus* CBS 115571 (Vesth et al., 2018), and isolated and characterized one new polyketide-non-ribosomal peptide hybrid molecule named pyranoviolin A (**1**). Furthermore, the biosynthetic gene cluster of **1** was readily identified in the genome of the fungus, and a plausible biosynthetic pathway of **1** has been proposed, suggesting the usefulness of the concept to obtain new natural products together with their biosynthetic information.

## Materials and Methods

### General

Organic solvents were purchased from Anaqua (Hong Kong) Co. Ltd., and other chemicals were purchased from Wako Chemicals Ltd., Thermo Fisher Scientific, Sigma-Aldrich, or J&K Scientific Ltd., unless noted otherwise. Oligonucleotide primers were purchased from Tech Dragon Limited. Polymerase chain reaction (PCR) was performed using a T100 Thermal Cycler (Bio-Rad Laboratories, Inc.) with the Phusion High-Fidelity DNA Polymerase (Thermo Fisher Scientific) or the Plant Direct PCR Kit (Vazyme Biotech Co., Ltd). Analytical LC/HR-ESI-MS analysis was performed on a Dionex Ultimate 3000 UHPLC system (Thermo Fisher Scientific) with a micrOTOF-Q II mass spectrometer (Bruker Daltonics), using a COSMOSIL 2.5Cholester packed column (2.0 i.d. × 100 mm; Nacalai Tesque, Inc.). Flash chromatography was performed using an Isolera Spektra One flash purification system (Biotage). Preparative HPLC was performed on a Waters 1525 Binary HPLC pump with a 2998 photodiode array detector (Waters Corporation), using an XBridge BEH C18 OBD Prep Column (100 Å, 5 μm, 19 i.d. × 250 mm; Waters Corporation). NMR spectra were obtained 600 MHz (^1^H)/150 MHz (^13^C)/60 MHz (^15^N) with a Bruker Ascend Avance III HD spectrometer, and chemical shifts were recorded with reference to solvent signals [^1^H NMR: DMSO-*d*_6_ 2.49 ppm; ^13^C NMR: DMSO-*d*_6_ 39.5 ppm; ^15^N NMR: 78.98 ppm (^15^N urea as an external reference)]. Optical rotations were measured with P-2000 Digital Polarimeter (JASCO Corporation). CD spectra were obtained with J-1500 Circular Dichroism Spectrophotometer (JASCO Corporation). X-ray diffraction data were collected on a Bruker D8 Venture Photon II diffractometer. The glufosinate solution for the fungal transformation was extracted from Basta (Bayer) as previously described (Chooi et al., 2010) and used at a 50 μL/mL concentration.

### HPLC Analysis

Analytical HPLC was performed with a solvent system of 20 mM formic acid (solvent A) and acetonitrile containing 20 mM formic acid (solvent B), at a flow rate of 0.4 mL/min and a column temperature of 40°C. The separation was performed using a linear gradient from 10:90 (solvent B/solvent A) to 100:0 for 10 min, 100:0 for the following 3 min, and a linear gradient from 100:0 to 10:90 within the following 2.5 min.

### Production and Purification of Pyranoviolin A (1)

*Aspergillus violaceofuscus* CBS 115571 was purchased from the Westerdijk Fungal Biodiversity Institute, inoculated on 100 YES agar plates [*ca.* 2 L; 20 g/L yeast extract, 150 g/L sucrose, 0.5 g/L MgSO_4_⋅7H_2_O, 20 g/L agar supplemented with 1mL/L of a trace element solution (10 g/L ZnSO_4_⋅7H_2_O, 5 g/L CuSO_4_⋅5H_2_O), pH 6.5], and cultivated for 9 days at 25°C. The resultant fungal cultures including agar were crushed into small pieces and extracted with ethyl acetate twice using an ultrasonic bath. The crude extract was subjected to flash chromatography with Biotage^®^ SNAP KP-Sil cartridge (100 g) and eluted stepwise using chloroform:ethyl acetate gradient (100:0 to 0:100). Fractions that contained **1** were concentrated and further purified by reverse-phase preparative HPLC (40% aqueous acetonitrile containing 0.05% trifluoroacetic acid, 10 mL/min) to yield 64.9 mg of colorless crystals.

### Calculation of the ECD Spectrum of Pyranoviolin A (1)

Initially, an exhaustive conformation space search of (7*R*)-**1** and (7*S*)-**1** were conducted by Conformer-Rotamer Ensemble Sampling Tool (CREST) version 2.8 (Pracht et al., 2020). Fifteen conformers for (7*R*)-**1** and (7*S*)-**1**, respectively, were obtained with relative energies in 5 kcal/mol. All conformers were then optimized in Gaussian 09 software package (Frisch et al., 2010) at M062x/def2tzvp level with PCM in methanol. The optimized conformers with the Boltzmann distribution >1% were further calculated for the ECD by using TDDFT at cam-b3lyp/tzvp level with PCM in methanol. The ECD spectrum was obtained by weighing the Boltzmann distribution rate of each geometric conformation in Multiwfn 3.6 (Lu and Chen, 2012).

The ECD spectrum is simulated by overlapping Gaussian functions for each transition according to:

$$\Delta \varepsilon \left(E\right)=\frac{1}{2.297\times 10^{-39}}\times \frac{1}{\sqrt{2\pi \sigma }}\sum_{i}^{A}\Delta E_{i}R_{i}{\text{e}}^{-{\left[\left(E-E_{i}\right)/\left(2\sigma \right)\right]}^{2}}$$

Where σ represents the width of the band at 1/*e* height, and Δ*E*_*i*_ and *R*_*i*_ are the excitation energies and rotational strengths for transition *i*, respectively. σ = 0.5 eV and *R*^*velocity*^ have been used in this work.

The optimized conformation geometries, thermodynamic parameters, key transitions, oscillator strengths, and rotatory strengths in the ECD spectrum and populations of all conformations were provided in Supplementary Tables 1–16.

### Gene Deletion Experiment of *pyvA*

The gene deletion of *pyvA* was performed by CRISPR-Cas9-mediated target gene break and repair with a microhomology repair template, as previously described for *Aspergillus fumigatus* (Al Abdallah et al., 2017). The components for the dual Cas9-gRNA system, namely Cas9 protein, CRISPR RNAs (crRNAs), and transactivating CRISPR RNA (tracrRNA), were purchased from Integrated DNA Technologies; two separate crRNAs were designed at 5′- and 3′-end regions of *pyvA*, respectively (protospacer sequences: 5′-AAAGGCACCACACAAGACGG-3′; 5′-CGGAACCAAGTCCACGACGA-3′). The Cas9 ribonucleoprotein (RNP) complexes were assembled as reported (Al Abdallah et al., 2017). The microhomology repair template for the fungal transformation was amplified from pBARI (Matsuda et al., 2014) using the primers pyvA_bar-F (5′-CCGGAATCTCCTTGGAGGAGATGAACGGCTCACGCACC TTTGGTGATTGGAATAACTGAC-3′) and pyvA_bar-R (5′-GGAGCGCCGTGAGATAGTTGACCGGAACCAAGTCC ACGACGTGACGATGAGCCGCTCTTG-3′), generating the glufosinate-resistant gene (*bar*) flanked by microhomology arms targeting *pyvA*. The transformation of *A. violaceofuscus* and selection of transformants were performed as previously described for *Aspergillus oryzae* (Matsuda et al., 2018) except that the lysing enzymes from *Trichoderma harzianum* (Sigma-Aldrich) was used as fungal cell lytic enzymes at a concentration of 40 mg/mL. The successful deletion of *pyvA* was confirmed by colony-direct PCR of the transformant using the primers pyvA-check-F (5′-CATCGAACAGAAGGATGTGAGGCGCAAAC-3′) and PptrA-check-R (5′-CTATCATCTGTTAGCCATTCCATCAACAGG-3′) (Supplementary Figure S10).

### Analytical Data

**Pyranoviolin A** (**1**): Colorless crystal; [α]^24^_*D*_ + 61.3 (*c* 1.00, MeOH); CD (*c* 0.025, MeOH) λ, nm (Δε) 200 (−5.9), 210 (−3.4), 219 (−4.1), 244 (+1.8), 271 (+4.6), 334 (−0.3); for NMR spectra see Supplementary Figures 1–7; HRMS (ESI) *m/z*: [M + Na]^+^ Calcd. for C_13_H_15_NO_5_Na 288.0842; Found 288.0850.

**Crystallographic data for pyranoviolin A** (**1**): C_13_H_15_NO_5_, *M* = 265.26, *a* = 9.7800(2) Å, *b* = 18.8599(3) Å, *c* = 20.9282(4) Å, α = 90°, β = 90°, γ = 90°, *V* = 3860.20(12) Å^3^, *T* = 213(2) K, space group *P*2_1_2_1_2_1_, *Z* = 12, μ(Cu Kα) = 0.893 mm^–1^, 43 655 reflections measured, 7883 independent reflections (*R*_*int*_ = 0.0435). The final *R*_1_ values were 0.0345 (*I* > 2σ(*I*)). The final *wR*(*F*^2^) values were 0.0918 (*I* > 2σ(*I*)). The final *R*_1_ values were 0.0389 (all data). The final *wR*(*F*^2^) values were 0.0961 (all data). The goodness of fit on *F*^2^ was 1.022. Flack parameter = 0.00(5). The crystallographic information file (CIF) for this crystal structure was submitted to The Cambridge Crystallographic Data Centre (CCDC), under reference number 2003775.

## Results and Discussion

### Search for a New Metabolite in *Aspergillus violaceofuscus* CBS 115571

**FIGURE 1:**
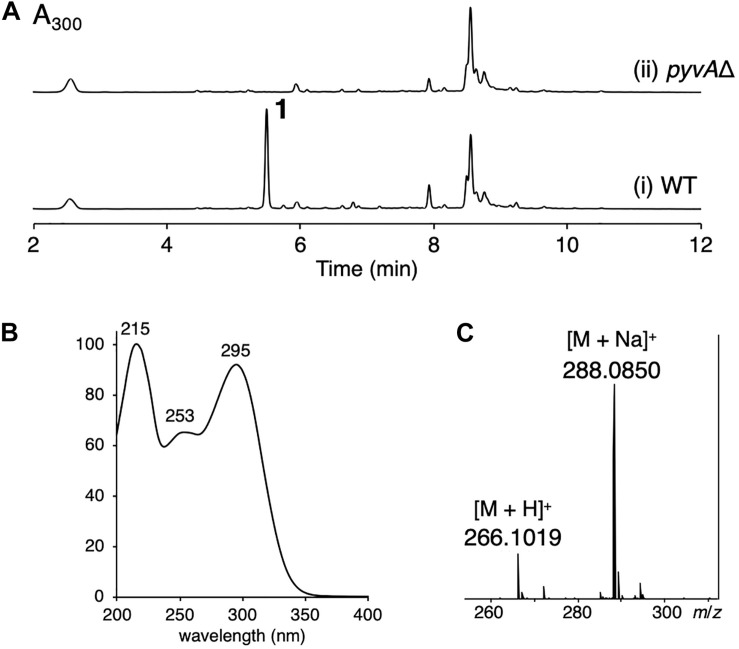
**(A)** HPLC profile of the ethyl acetate extract of (i) the wild type (WT) and (ii) the *pyvA*Δ mutant of *A. violaceofuscus* CBS 115571. The chromatograms were monitored at 300 nm. **(B)** The UV spectrum of **1**. **(C)** The mass spectrum of **1**.

*Aspergillus violaceofuscus* CBS 115571 is one of the *Aspergillus* fungi whose genomes have been recently sequenced and published (Vesth et al., 2018). Analysis of the genome of the *A. violaceofuscus* strain using antiSMASH (Blin et al., 2019) suggested that the fungus harbors ∼80 biosynthetic gene clusters for secondary metabolites; however, only a few natural products have been isolated from this species (Myobatake et al., 2014; Liu et al., 2018), implying the potential of *A. violaceofuscus* to produce new natural products. The fungus produced several major metabolites, which are predicted to be calbistrins (Brill et al., 1993), eupenoxide (Mehta and Roy, 2004), and himeic acid A (Tsukamoto et al., 2005) based on their molecular formulas and UV spectra (Supplementary Figure 8). The ethyl acetate extract of the fungus cultivated on YES agar plate revealed the presence of one major product **1** (Figure 1A, trace i). Compound **1** exhibited UV maxima at 215 nm, 253 nm, and 295 nm (Figure 1B), and its molecular formula was determined to be C_13_H_15_NO_5_ by HR-ESI-MS analysis (Figure 1C). The database search indicated that **1** is a previously unreported metabolite. Thus, the *A. violaceofuscus* strain was cultivated on a large scale, and **1** was successfully purified by a series of chromatographic procedures.

### Characterization of Pyranoviolin A

As mentioned above, the molecular formula of **1** was established as C_13_H_15_NO_5_, indicating seven degrees of unsaturation. The ^13^C NMR spectrum revealed 13 signals (Table 1), consisting of two methyls, including one methoxy (C-8), two methylenes, three methines, including one oxymethine (C-7) and two olefinic methines (C-1′ and C-2′), four olefinic quaternary carbons (C-2, C-3, C-4a, C-7a), and two carbonyls (C-4 and C-5). Additionally, interpretation of ^1^H and ^15^N NMR spectra illuminated the presence of one hydroxy group (7-OH: δ_*H*_ 6.81) and one amide nitrogen (6-NH: δ_*H*_ 8.63; δ_*N*_ 129.2). Altogether, it was indicated that **1** possesses a bicyclic structure.

**TABLE 1:** NMR data of **1**.

Position	δ_*C*_, type	δ_*N*_	δ_*H*_, mult. (*J* in Hz)
2	154.1, C		
3	143.7, C		
4	169.9, C		
4a	113.9, C		
5	164.6, C		
6-NH		129.2	8.63, brs
7	74.9, CH		5.73, dd (9.2, 1.5)
7a	174.8, C		
8	60.3, CH_3_		3.76, s
1′	117.2, CH		6.57, d (15.8)
2′	139.7, CH		6.61, dd (15.8, 5.9)
3′	34.5, CH_2_		2.27, m
4′	21.4, CH_2_		1.48, sext (7.3)
5′	13.6, CH_3_		0.91, t (7.3)
7-OH			6.81, d (9.2)

*^1^H NMR, 600 MHz; ^13^C NMR, 150 MHz; ^15^N NMR, 60 MHz (in DMSO-d_6_).*

**FIGURE 2:**
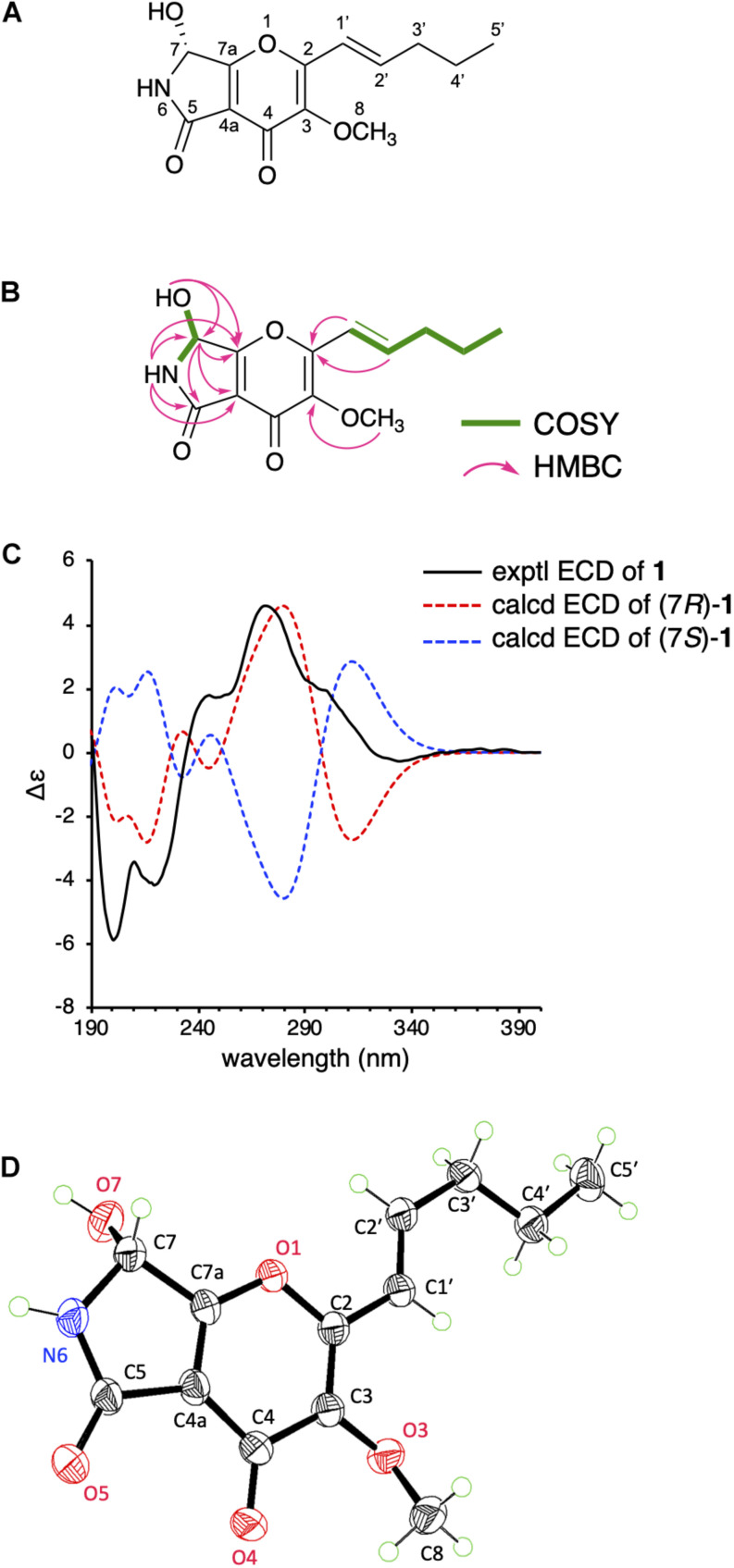
**(A)** The structure of **1**. **(B)** ^1^H-^1^H COSY and key HMBC correlations in **1**. **(C)** Experimental and calculated ECD spectra of **1**. **(D)** Single-crystal ORTEP diagram of **1**.

The ^1^H-^1^H COSY spectrum then revealed the spin systems of 6-NH/H-7/7-OH and H-1′/H-2′/H_2_-3′/H_2_-4′/H_3_-5′ (Figures 2A,B). Furthermore, the HMBC correlations of H-1′ (δ_*H*_ 6.57) and H-2′ (δ_*H*_ 6.61) to C-2, 6-NH (δ_*H*_ 8.63) to C-4a, C-5, C-7, and C-7a, H-7 (δ_*H*_ 5.73) to C-4a, C-5, and C-7a, 7-OH (δ_*H*_ 6.81) to C-7 and C-7a, and H-8 (δ_*H*_ 3.76) to C-3 established the connections of C-1′ to C-2, C-5 to C-4a and N-6, C-7a to C-7 and C-4a, and C-3 to C-8 via one oxygen atom (Figure 2B). In the course of the structural determination, we noted that the NMR spectra of **1** highly resemble those of another fungal metabolite pyranonigrin F isolated from *Penicillium brocae* (Meng et al., 2015), except that the signal for the methoxy group is missing in pyranonigrin F [Note that two distinct natural products are individually named “pyranonigrin F” (Meng et al., 2015; Yamamoto et al., 2015) (Supplementary Figure 9)]. The comparison of the NMR spectra elucidated the presence of γ-pyrone in **1**, thus establishing the planar structure of **1**, which is a methylated analog of pyranonigrin F and hereby named pyranoviolin A. Pyranoviolin A is the first example of a pyranonigrin analogs with the C-3 methoxy group.

To determine the absolute configuration of **1** at C-7 position, we obtained the calculated electron circular dichroism (ECD) spectra of (7*R*)- and (7*S*)-**1** and compared them with the experimental ECD spectrum of **1** (Figure 2C), which suggested the absolute configuration of **1** to be 7*R*. To further confirm the predicted structure, we performed single-crystal X-ray diffraction analysis of **1** using Cu Kα radiation and successfully determined the crystal structure of **1** with a Flack parameter of 0.00(5) (Figure 2D), which is consistent with the structure deduced from the NMR data and the calculation. Collectively, the structure of **1** has been unambiguously established, revealing that **1** possesses the same absolute stereochemistry as those of pyranonigrins A and F.

We then investigated the biological activity of **1**. Since the demethylated analog of **1**, pyranonigrin F, reportedly displays potent activity against some bacteria (Meng et al., 2015), including *Staphylococcus aureus*, we evaluated the antibacterial property of **1** using the previously described method (Zhao et al., 2018). However, **1** unfortunately did not show any antibacterial activity against the six tested strains, namely *Staphylococcus aureus* ATCC 6538, *Pseudomonas aeruginosa* ATCC 7700, *Escherichia coli* ATCC 10536, *Staphylococcus epidermidis* ATCC 12228, *Bacillus cereus*, and *Salmonella typhimurium* TA100. Given that the structural difference of **1** from pyranonigrin F is only the presence of the methyl group, it could be reasoned that the C-3 hydroxy group is critical for the biological activity.

### Investigation on the Biosynthesis of Pyranoviolin A

**FIGURE 3:**
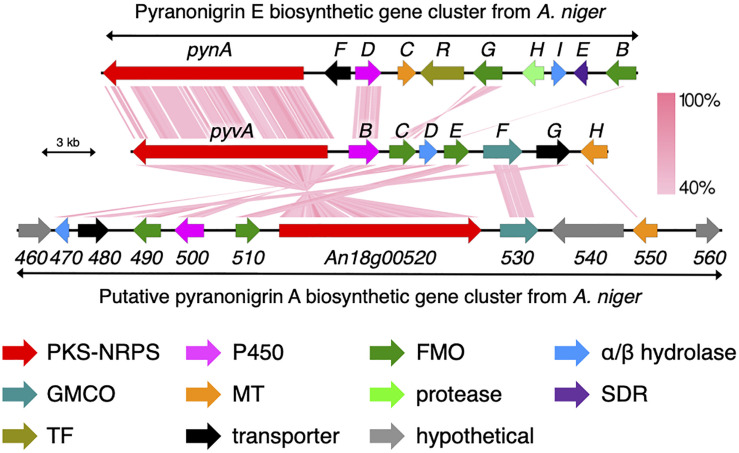
Schematic representation of the *pyv* cluster and tblastx comparison with the biosynthetic gene clusters for pyranonigrins A and E. PKS-NRPS, polyketide synthase-non-ribosomal peptide synthetase; P450, cytochrome P450 monooxygenase; FMO, flavin-dependent monooxygenase; GMCO, glucose–methanol–choline family of oxidoreductase; MT, methyltransferase; SDR, short-chain dehydrogenase/reductase; TF, transcription factor.

We next sought to identify the biosynthetic gene cluster of pyranoviolin A (**1**) in the genome of *A. violaceofuscus* CBS 115571. Given the structural similarity of **1** and pyranonigrins, the biosynthetic gene cluster of **1** should be somewhat homologous to those of pyranonigrins (Awakawa et al., 2013; Yokoyama et al., 2017; Tang et al., 2018). Examination of the genome sequence identified one candidate gene cluster for the pyranoviolin A biosynthesis (Figure 3 and Table 2), which was designated as the *pyv* cluster (DDBJ/EMBL/GenBank accession number: BR001648). The *pyv* cluster encodes a polyketide synthase (PKS)-non-ribosomal peptide synthetase (NRPS) hybrid PyvA, a cytochrome P450 monooxygenase PyvB, two flavin-dependent monooxygenases (FMOs) PyvC and PyvE, an α/β hydrolase PyvD, glucose–methanol–choline (GMC) family of oxidoreductase PyvF, and *O*-methyltransferase PyvH. Among these proteins, homologous enzymes of PyvA, PyvB, and PyvC are involved in the biosynthesis of pyranonigrins A and E (Figure 3), which are known to be responsible for the construction of the backbone skeleton of pyranonigrins (Yamamoto et al., 2015; Tang et al., 2018). Meanwhile, close homologs of PyvD, PyvE, and PyvF are encoded by the biosynthetic gene clusters of pyranonigrin A; PyvD might be engaged in the Dieckmann condensation to release the polyketide-non-ribosomal peptide chain, whereas the PyvE and PyvF homologs in the pyranonigrin A pathway have no apparent function in the biosynthesis. The *O*-methyltransferase gene *pyvH* is specifically found in the *pyv* cluster, which is consistent with the observation that C-3 methoxy group is only present in pyranoviolin A (**1**).

**TABLE 2:** Annotation of each gene in the *pyv* cluster.

Gene (Accession code)	Amino acid (Base pairs)	Protein homolog [origin]	Similarity/identity (%)	Proposed function
*pyvA* (PYI13694.1)	3940 (11971)	ANI_1_982164 [*Aspergillus niger*]	73/60	Polyketide synthase-non-ribosomal peptide synthetase
*pyvB* (PYI13693.1)	487 (1644)	ANI_1_978164 [*Aspergillus niger*]	78/65	Cytochrome P450 monooxygenase
*pyvC* (PYI13692.1)	427 (1603)	ANI_1_976164 [*Aspergillus niger*]	84/70	Flavin-dependent monooxygenase
*pyvD* (PYI13691.1)	245 (934)	ANI_1_972164 [*Aspergillus niger*]	79/67	α/β hydrolase
*pyvE* (PYI13690.1)	452 (1431)	ANI_1_980164 [*Aspergillus niger*]	83/74	Flavin-dependent monooxygenase
*pyvF* (PYI13689.1)	629 (2323)	ANI_1_984164 [*Aspergillus niger*]	62/45	Glucose-methanol-choline family oxidoreductase
*pyvG* (PYI13688.1)	543 (1894)	ANI_1_974164 [*Aspergillus niger*]	78/65	Major facilitator superfamily transporter
*pyvH* (PYI13687.1)	426 (1559)	PaMT [*Diaporthe amygdali*]	55/36	*O*-methyltransferase

To confirm the involvement of the *pyv* cluster in the biosynthesis of pyranoviolin A (**1**), we deleted the PKS-NRPS hybrid gene *pyvA* by means of CRISPR-Cas9-based genome editing technology (Al Abdallah et al., 2017). The disruption of *pyvA* completely abolished the production of **1** (Figure 1A, trace ii), thus demonstrating that the *pyv* cluster is indeed responsible for the pyranoviolin A biosynthesis.

**FIGURE 4:**
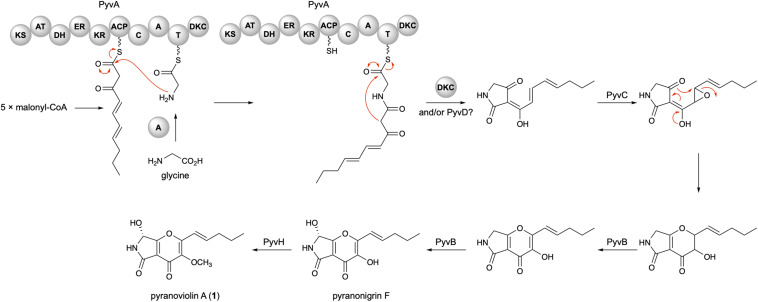
Predicted biosynthetic pathway of **1**.

On the basis of the bioinformatic analysis and previous biosynthetic studies on pyranonigrins, the biosynthetic route leading to pyranoviolin A (**1**) can be proposed as follows (Figure 4). Initially, the PKS portion of PyvA synthesizes C_10_ carbon chain from five molecules of malonyl-CoA, which is then condensed with the thiolation (T) domain-bound glycine activated by the adenylation (A) domain. The subsequent chain release by Dieckmann condensation (DKC) could be catalyzed by the DKC domain present at the C-terminus of PyvA and/or the α/β hydrolase PyvD, installing the tetramic acid moiety. The FMO PyvC next epoxidizes one of the olefins of the polyketide part, and the epoxide ring-opening induces the dihydro-γ-pyrone ring formation. The P450 PyvB would be responsible for the two consecutive reactions, in which the dihydro-γ-pyrone is oxidized to γ-pyrone and C-7 is hydroxylated to yield pyranonigrin F. Finally, the *O*-methyltransferase PyvH, which is specifically found in the pyranoviolin A pathway, methylates the C-3 hydroxy group to complete the biosynthesis.

## Conclusion

In this study, we discovered a new polyketide-non-ribosomal peptide hybrid molecule pyranoviolin A (**1**) from *Aspergillus violaceofuscus* CBS 115571 and identified the biosynthetic gene cluster of **1** in the genome of the fungus. Compound **1** possesses a methoxy group at C-3, which had not been reported in this family of natural products. Our study indicates that working on genome-sequenced microorganisms whose metabolic profiles have not, or poorly, been investigated can readily discover new natural products and link the metabolites with their biosynthetic gene clusters. Given that many microbial genomes have been or being sequenced, this approach would accelerate the discovery of new natural products and characterization of their biosynthetic enzymes, which expands the natural product diversity and repertoire of unusual biosynthetic enzymes.

## Data Availability Statement

The datasets presented in this study can be found in online repositories. The names of the repository/repositories and accession number(s) can be found in the article/Supplementary Material.

## Author Contributions

XW, LC, and YM performed the experiments and analyzed the data. J-WT performed the ECD calculation. YM designed the research and wrote the manuscript. All authors approved the manuscript.

## Conflict of Interest

The authors declare that the research was conducted in the absence of any commercial or financial relationships that could be construed as a potential conflict of interest.

## References

[B1] Al Abdallah Q. Ge W. Fortwendel J. R. (2017). A simple and universal system for gene manipulation in *Aspergillus fumigatus*: *in vitro*-assembled Cas9-guide RNA ribonucleoproteins coupled with microhomology repair templates. *mSphere* 2:e00446-17. 10.1128/mSphere.00446-17 29202040 PMC5700375

[B2] Awakawa T. Yang X.-L. Wakimoto T. Abe I. (2013). Pyranonigrin E: a PKS-NRPS hybrid metabolite from *Aspergillus niger* identified by genome mining. *ChemBioChem* 14 2095–2099. 10.1002/cbic.201300430 24106156

[B3] Blin K. Shaw S. Steinke K. Villebro R. Ziemert N. Lee S. Y. (2019). antiSMASH 5.0: updates to the secondary metabolite genome mining pipeline. *Nucleic Acids Res.* 47 W81–W87. 10.1093/nar/gkz310 31032519 PMC6602434

[B4] Brakhage A. A. Schroeckh V. (2011). Fungal secondary metabolites–strategies to activate silent gene clusters. *Fungal Genet. Biol.* 48 15–22. 10.1016/j.fgb.2010.04.004 20433937

[B5] Brill G. M. Chen R. H. Rasmussen R. R. Whittern D. N. Mcalpine J. B. (1993). Calbistrins, novel antifungal agents produced by *Penicillium restrictum*. II. Isolation and elucidation of structure. *J. Antibiot.* 46 39–47. 10.7164/antibiotics.46.39 8436558

[B6] Cacho R. A. Tang Y. Chooi Y.-H. (2015). Next-generation sequencing approach for connecting secondary metabolites to biosynthetic gene clusters in fungi. *Front. Microbiol.* 5:774. 10.3389/fmicb.2014.00774 25642215 PMC4294208

[B7] Chooi Y.-H. Cacho R. Tang Y. (2010). Identification of the viridicatumtoxin and griseofulvin gene clusters from *Penicillium aethiopicum*. *Chem. Biol.* 17 483–494. 10.1016/j.chembiol.2010.03.015 20534346 PMC2884005

[B8] Frisch M. J. Trucks G. W. Schlegel H. B. Scuseria G. E. Robb M. A. Cheeseman J. R. (2010). *Gaussian 09, Revision E.01.* Wallingford, CT: Gaussian Inc.

[B9] Liu J. Gu B. Yang L. Yang F. Lin H. (2018). New anti-inflammatory cyclopeptides from a sponge-derived fungus *Aspergillus violaceofuscus*. *Front. Chem.* 6:226. 10.3389/fchem.2018.00226 29963550 PMC6010530

[B10] Lu T. Chen F. (2012). Multiwfn: a multifunctional wavefunction analyzer. *J. Comput. Chem.* 33 580–592. 10.1002/jcc.22885 22162017

[B11] Matsuda Y. Bai T. Phippen C. B. W. Nødvig C. S. Kjærbølling I. Vesth T. C. (2018). Novofumigatonin biosynthesis involves a non-heme iron-dependent endoperoxide isomerase for orthoester formation. *Nat. Commun.* 9:2587. 10.1038/s41467-018-04983-2 29968715 PMC6030086

[B12] Matsuda Y. Wakimoto T. Mori T. Awakawa T. Abe I. (2014). Complete biosynthetic pathway of anditomin: nature’s sophisticated synthetic route to a complex fungal meroterpenoid. *J. Am. Chem. Soc.* 136 15326–15336. 10.1021/ja508127q 25216349

[B13] Mehta G. Roy S. (2004). Enantioselective total synthesis of (+)-eupenoxide and (+)-phomoxide: revision of structures and assignment of absolute configuration. *Org. Lett.* 6 2389–2392. 10.1021/ol0492288 15228286

[B14] Meng L.-H. Li X.-M. Liu Y. Wang B.-G. (2015). Polyoxygenated dihydropyrano[2,3-c]pyrrole-4,5-dione derivatives from the marine mangrove-derived endophytic fungus *Penicillium brocae* MA-231 and their antimicrobial activity. *Chin. Chem. Lett.* 26 610–612. 10.1016/j.cclet.2015.01.024

[B15] Montiel D. Kang H.-S. Chang F.-Y. Charlop-Powers Z. Brady S. F. (2015). Yeast homologous recombination-based promoter engineering for the activation of silent natural product biosynthetic gene clusters. *Proc. Natl. Acad. Sci. U.S.A.* 112 8953–8958. 10.1073/pnas.1507606112 26150486 PMC4517240

[B16] Myobatake Y. Takemoto K. Kamisuki S. Inoue N. Takasaki A. Takeuchi T. (2014). Cytotoxic alkylated hydroquinone, phenol, and cyclohexenone derivatives from *Aspergillus violaceofuscus* Gasperini. *J. Nat. Prod.* 77 1236–1240. 10.1021/np401017g 24786915

[B17] Pracht P. Bohle F. Grimme S. (2020). Automated exploration of the low-energy chemical space with fast quantum chemical methods. *Phys. Chem. Chem. Phys.* 22 7169–7192. 10.1039/C9CP06869D 32073075

[B18] Rutledge P. J. Challis G. L. (2015). Discovery of microbial natural products by activation of silent biosynthetic gene clusters. *Nat. Rev. Microbiol.* 13:509. 10.1038/nrmicro3496 26119570

[B19] Tang M.-C. Zou Y. Yee D. Tang Y. (2018). Identification of the pyranonigrin A biosynthetic gene cluster by genome mining in *Penicillium thymicola* IBT 5891. *AIChE J.* 64 4182–4186. 10.1002/aic.16324 31588145 PMC6777573

[B20] Tsukamoto S. Hirota H. Imachi M. Fujimuro M. Onuki H. Ohta T. (2005). Himeic acid A: a new ubiquitin-activating enzyme inhibitor isolated from a marine-derived fungus, *Aspergillus* sp. *Bioorgan. Med. Chem. Lett.* 15 191–194. 10.1016/j.bmcl.2004.10.012 15582438

[B21] Vesth T. C. Nybo J. L. Theobald S. Frisvad J. C. Larsen T. O. Nielsen K. F. (2018). Investigation of inter- and intraspecies variation through genome sequencing of *Aspergillus* section *Nigri*. *Nat. Genet.* 50 1688–1695. 10.1038/s41588-018-0246-1 30349117

[B22] Yamamoto T. Tsunematsu Y. Noguchi H. Hotta K. Watanabe K. (2015). Elucidation of pyranonigrin biosynthetic pathway reveals a mode of tetramic acid, fused γ-pyrone, and *exo*-methylene formation. *Org. Lett.* 17 4992–4995. 10.1021/acs.orglett.5b02435 26414728

[B23] Yan Y. Liu N. Tang Y. (2020). Recent developments in self-resistance gene directed natural product discovery. *Nat. Prod. Rep.* 37 879–892. 10.1039/C9NP00050J 31912842 PMC7340575

[B24] Yokoyama M. Hirayama Y. Yamamoto T. Kishimoto S. Tsunematsu Y. Watanabe K. (2017). Integration of chemical, genetic, and bioinformatic approaches delineates fungal polyketide–peptide hybrid biosynthesis. *Org. Lett.* 19 2002–2005. 10.1021/acs.orglett.7b00559 28361537

[B25] Zarins-Tutt J. S. Barberi T. T. Gao H. Mearns-Spragg A. Zhang L. Newman D. J. (2016). Prospecting for new bacterial metabolites: a glossary of approaches for inducing, activating and upregulating the biosynthesis of bacterial cryptic or silent natural products. *Nat. Prod. Rep.* 33 54–72. 10.1039/C5NP00111K 26538321

[B26] Zhao Z.-Z. Zhao K. Chen H.-P. Bai X. Zhang L. Liu J.-K. (2018). Terpenoids from the mushroom-associated fungus *Montagnula donacina*. *Phytochemistry* 147 21–29. 10.1016/j.phytochem.2017.12.015 29287257

