# Supplementary material for: Discovery of Pyranoviolin A and Its Biosynthetic Gene Cluster in Aspergillus violaceofuscus
Source: Front Microbiol. 2020 Oct 7;11:562063. doi: 10.3389/fmicb.2020.562063 (PMC7575713; doi:10.3389/fmicb.2020.562063)
Supplement: Supplementary file 1 [file Data_Sheet_1.PDF]

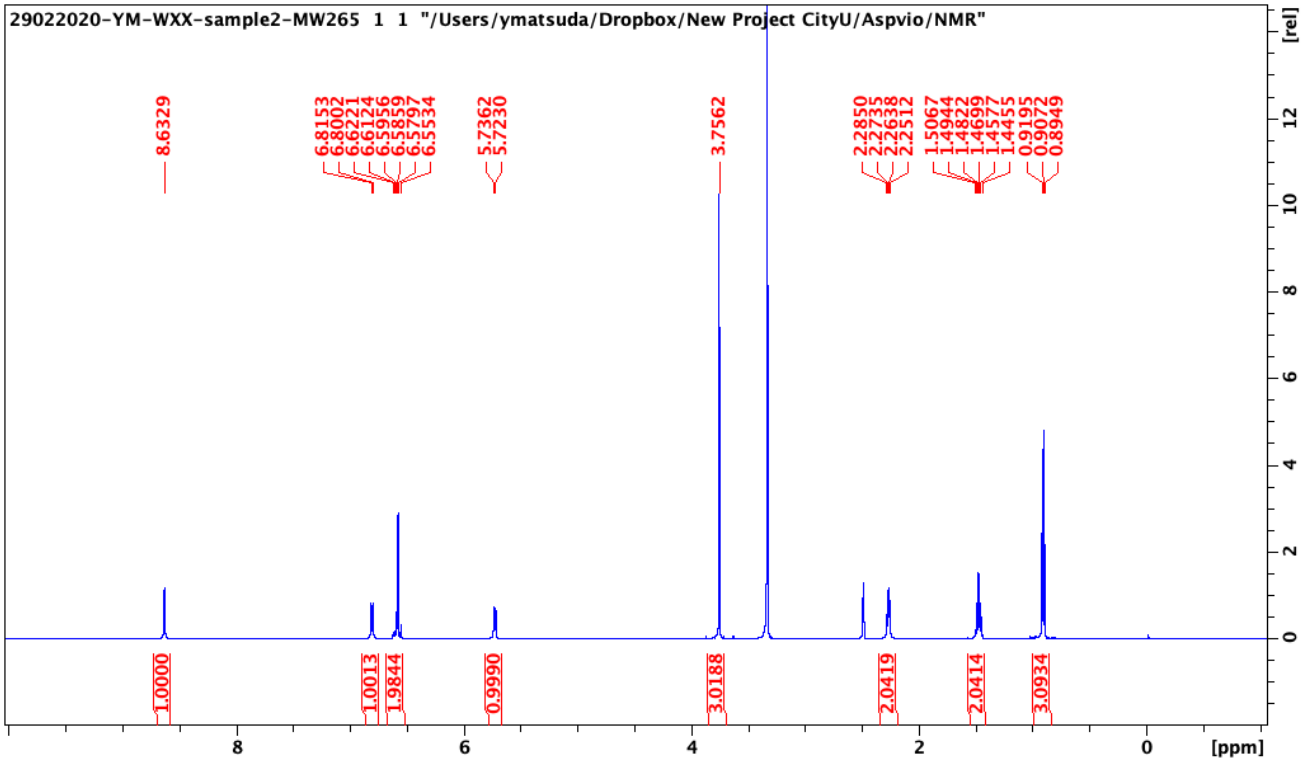

Supplementary Figure 1. ^{1}H NMR spectrum of pyranoviolin A (**1**).

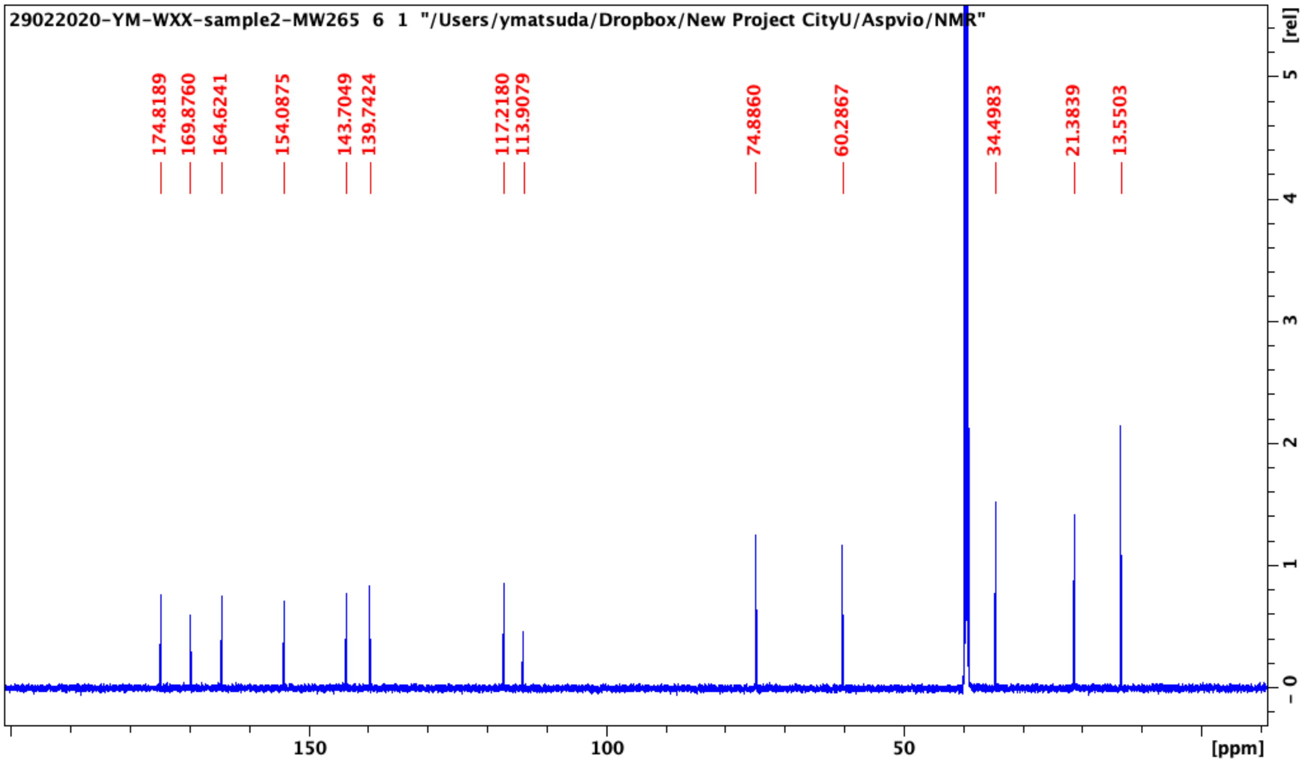

Supplementary Figure 2. ^{13}C NMR spectrum of pyranoviolin A (**1**).

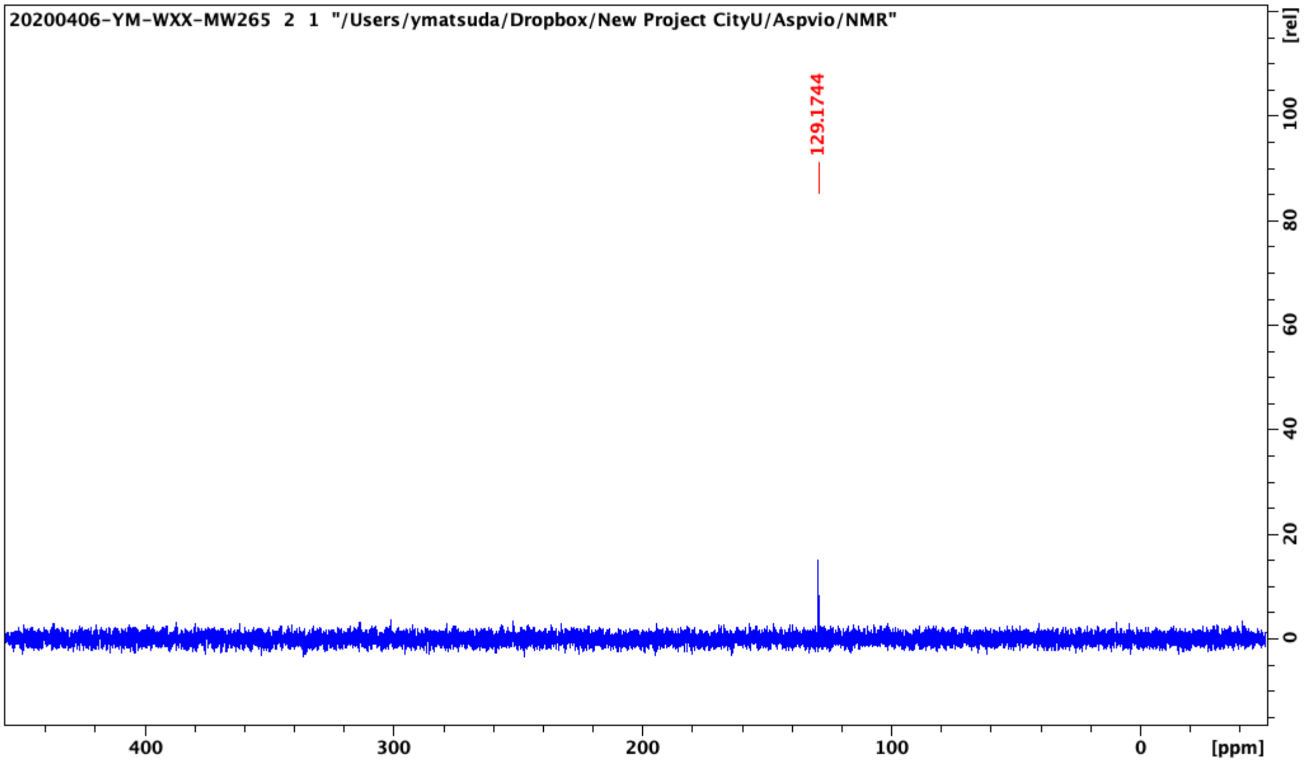

Supplementary Figure 3. ^{15}N NMR spectrum of pyranoviolin A (**1**).

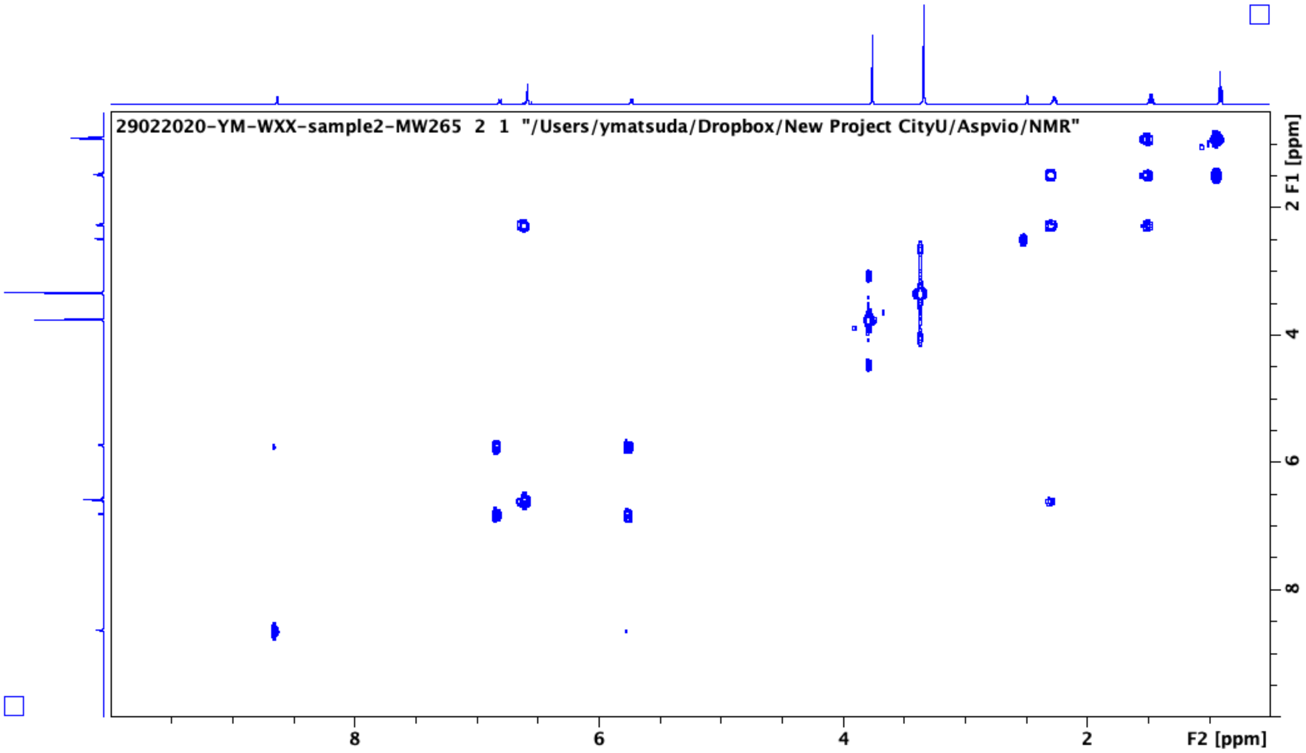

Supplementary Figure 4. ^{1}H-^{1}H COSY spectrum of pyranoviolin A (**1**).

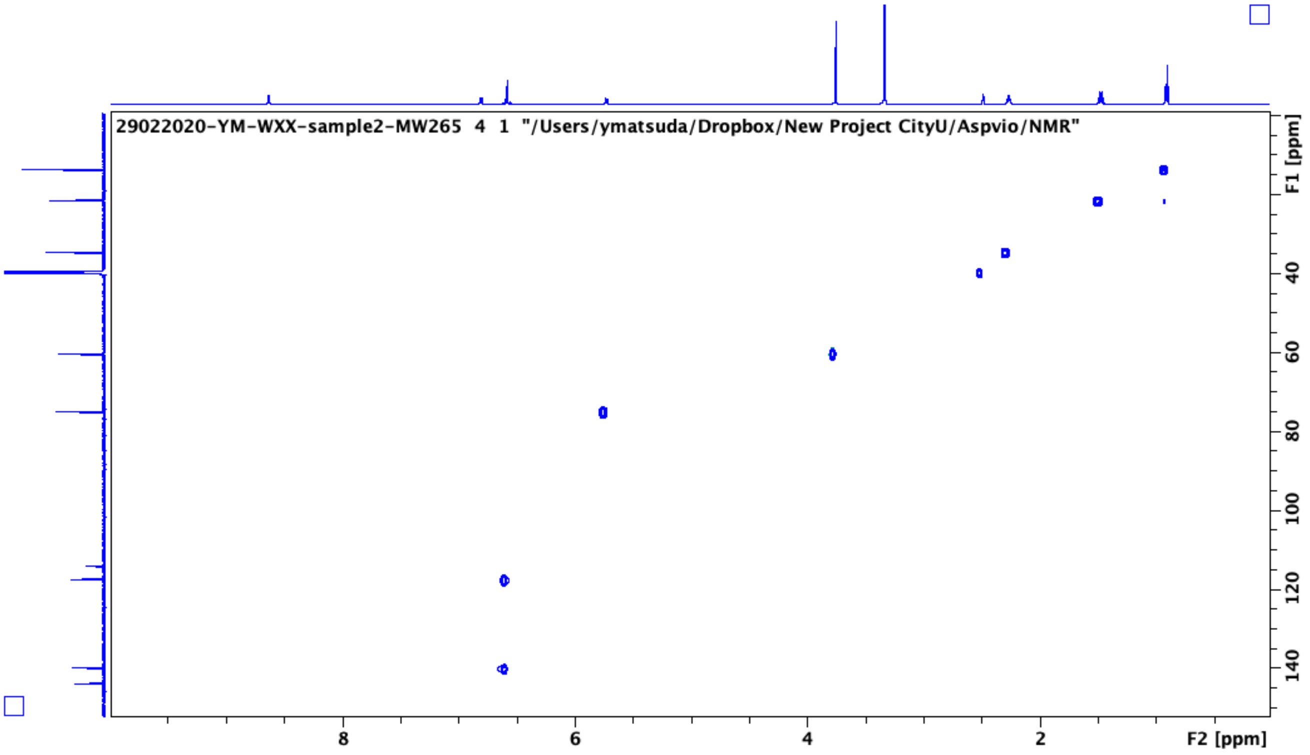

Supplementary Figure 5. HSQC spectrum of pyranoviolin A (**1**).

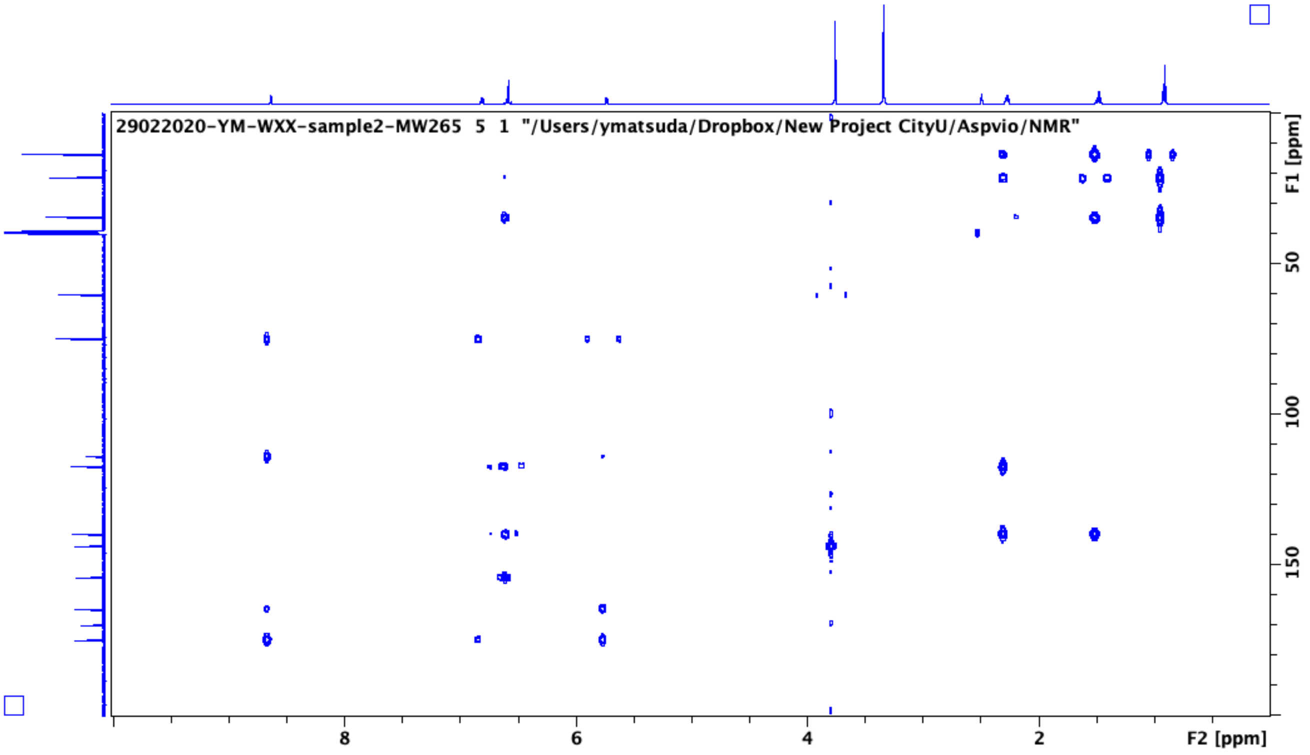

Supplementary Figure 6. HMBC spectrum of pyranoviolin A (**1**).

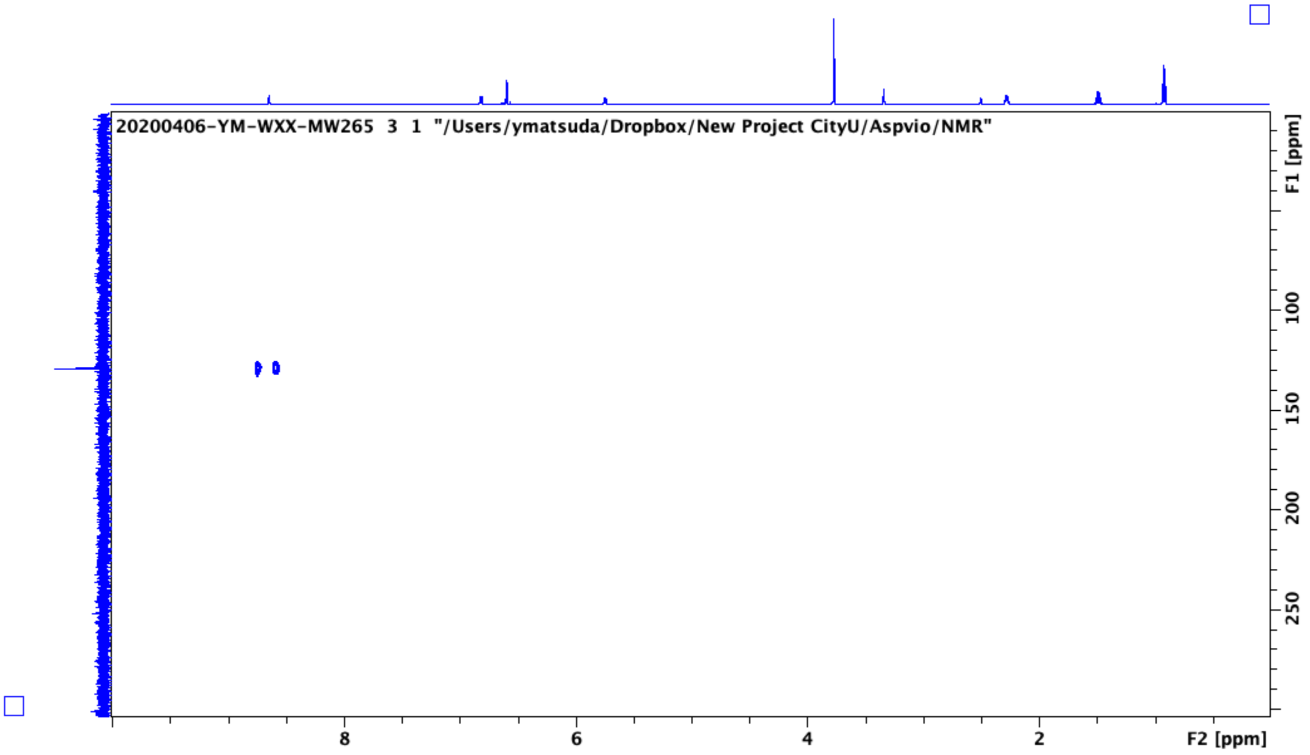

Supplementary Figure 7. ^{1}H-^{15}N HMBC spectrum of pyranoviolin A (**1**).

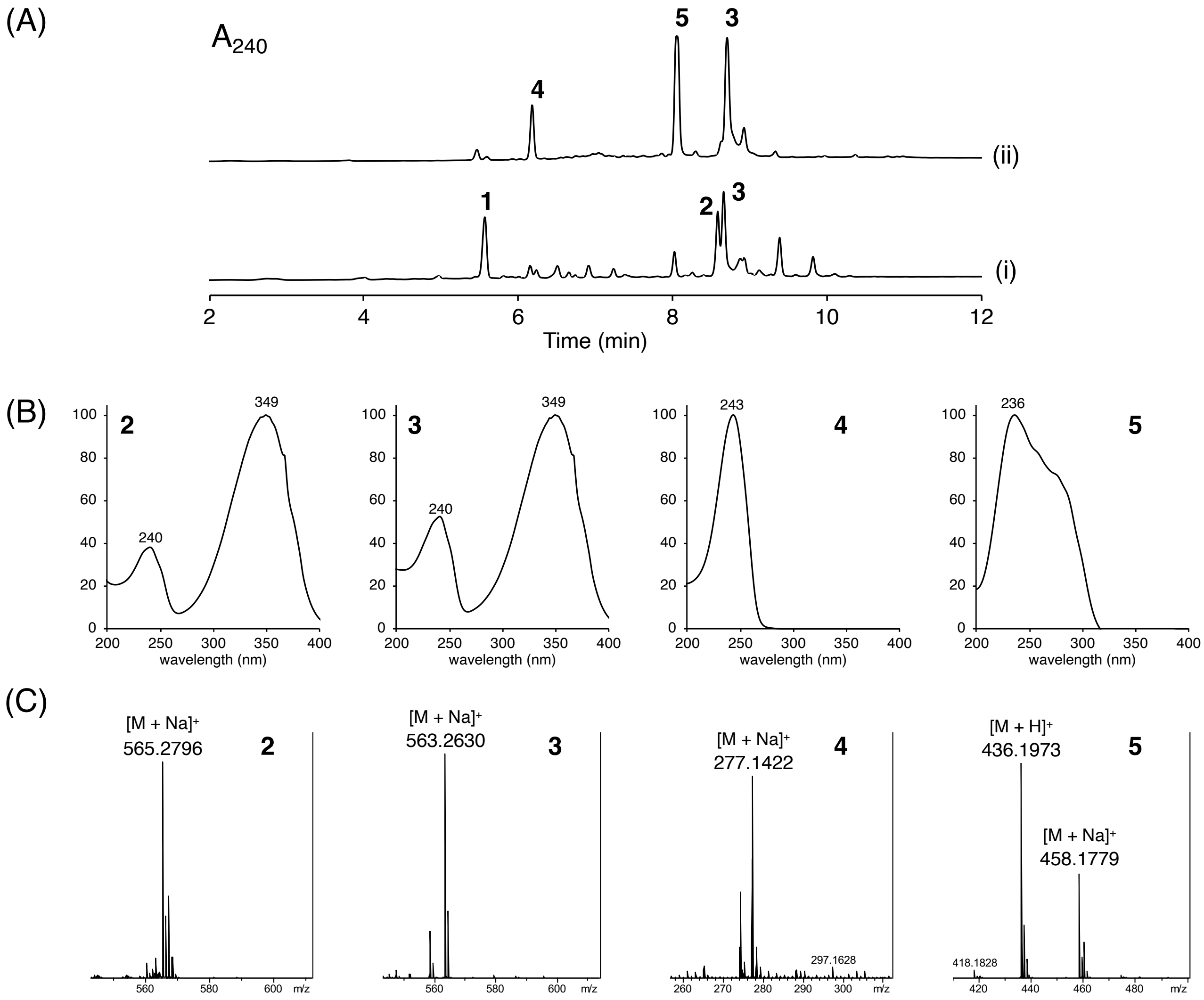

Supplementary Figure 8. (**A**) HPLC profiles of the ethyl acetate extract of *A. violaceofuscus* CBS 115571 cultivated on (i) YES agar medium and (ii) malt extract agar (MEA) medium. The chromatogram was monitored at 240 nm. (**B**) The UV spectra of the major metabolites of *A. violaceofuscus* CBS 115571, **2**-**5**. (**C**) The mass spectrum of **2**-**5**. Compounds **2**-**5** are predicted to be calbistrin C, calbistrin A, eupenoxide, and himeic acid A, respectively, based on their molecular formulas and UV spectra.

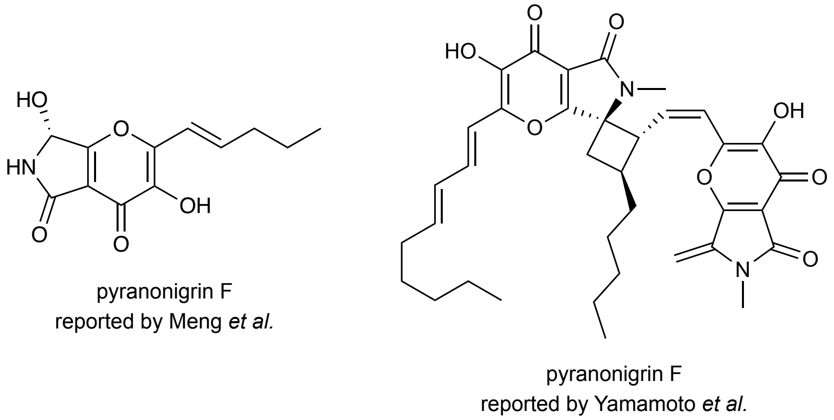

Supplementary Figure 9. Structures of two natural products named pyranonigrin F.

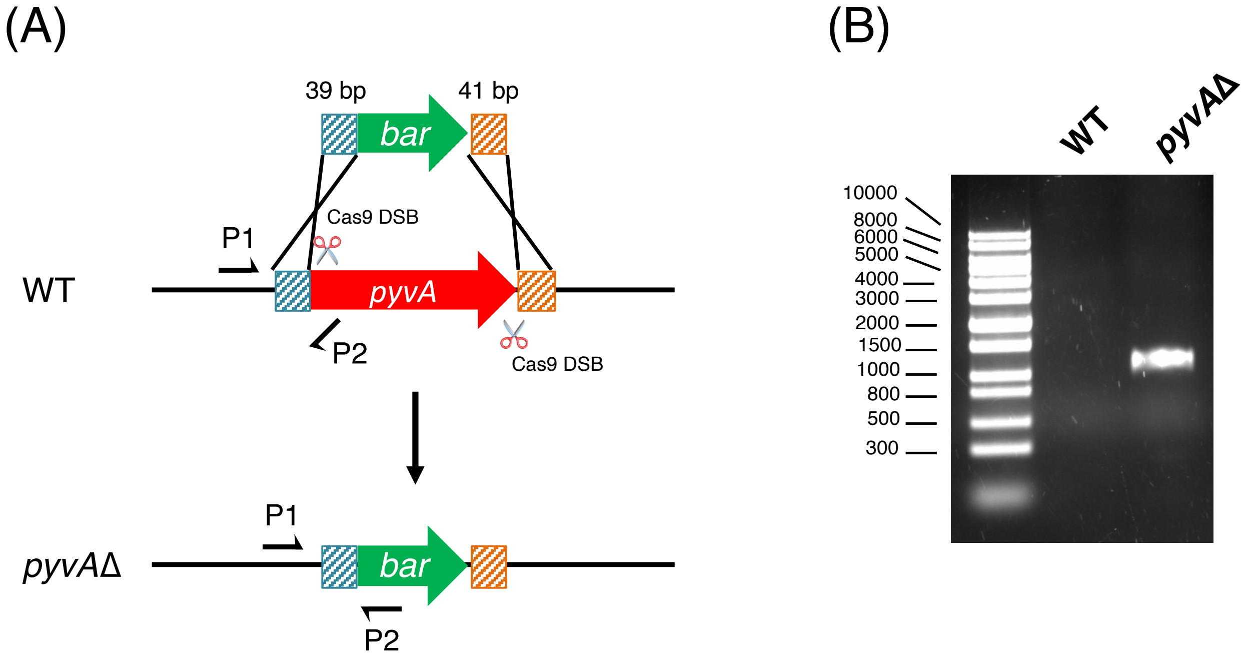

Supplementary Figure 10. Deletion of the PKS-NRPS gene *pyvA*. (A) Procedure for the deletion of *pyvA* using *bar* (glufosinate resistant gene) as a selection marker. The scissors indicate the positions at which DNA double-strand breaks (DSBs) occur by Cas9. (B) Result of diagnostic PCR performed with the two primers P1 (pyvA-check-F), binding outside of the homology arm at the 5′ end of *pyvA*, and P2 (PptrA-check-R), binding to the promoter region of *bar* (see the Materials and Methods section for the primer sequences). Since P2 does not bind to the wild type genome, a band is only amplified upon successful deletion of the targeted gene.

Supplementary Table 1. Conformational analysis of the M062x/def2tzvp level optimized conformers with boltzmann distribution > 1% of (7*R*)-1 in the IEFPCM model (methanol) (T=298.15 K)

Conformer	C (Hartree)	G (kcal/mol)	ΔG (kcal/mol)	Population
(7*R*)-1-a	0.2232	-586737.239188	0.0	44.39%
(7*R*)-1-b	0.223515	-586736.87963	0.359557	24.18%
(7*R*)-1-c	0.224221	-586736.624865	0.614322	15.73%
(7*R*)-1-d	0.223542	-586736.12914	1.110047	6.81%
(7*R*)-1-e	0.224376	-586735.80535	1.433837	3.94%
(7*R*)-1-f	0.224831	-586735.64973	1.589458	3.03%
(7*R*)-1-g	0.224066	-586735.38116	1.858027	1.92%

Supplementary Table 2. Conformational analysis of the M062x/def2tzvp level optimized conformers with boltzmann distribution > 1% of (7*R*)-1 in the IEFPCM model (methanol) (T=298.15 K)

Conformer	C (Hartree)	G (kcal/mol)	ΔG (kcal/mol)	Population
(7*S*)-1-a	0.223206	-586737.234795	0.0	51.74%
(7*S*)-1-b	0.223523	-586736.87461	0.360185	28.16%
(7*S*)-1-c	0.224425	-586736.50062	0.734175	14.97%
(7*S*)-1-d	0.224831	-586735.64973	1.585065	3.56%
(7*S*)-1-e	0.221392	-586735.16781	2.066985	1.58%

Supplementary Table 3. Atomic coordinates (Å) of conformers with boltzmann distribution > 1% of (7*R*)-**1** obtained at the M062x/def2tzvp level of theory in the IEFPCM model (methanol) (T=298.15 K)

(7*R*)-**1**-a							
C	1.624518	0.885083	-0.092160	O	-0.482824	-2.453442	0.741516
C	2.361149	-0.206739	0.159982	C	-0.530048	-3.434192	-0.299189
C	1.722170	-1.477638	0.457030	H	2.324995	2.487234	-1.357668
C	0.255169	-1.357738	0.428285	H	4.616859	2.046348	-0.424947
C	-0.386821	-0.191488	0.155849	H	2.308933	2.913532	1.419549
O	0.309827	0.956381	-0.101578	H	-2.380669	-0.942638	0.357679
C	2.463354	2.116364	-0.340366	H	-1.848722	1.975617	-0.410969
N	3.780370	1.559031	-0.143284	H	-4.186080	2.119245	0.437344
C	3.792217	0.204607	0.081963	H	-4.186815	1.649928	-1.235303
O	2.293480	-2.525364	0.710565	H	-4.522946	-0.747384	-0.570894
O	4.779776	-0.485997	0.182421	H	-4.520283	-0.272982	1.117212
O	2.153965	3.183021	0.504400	H	-6.857986	-0.503409	0.278979
C	-1.829692	-0.043153	0.120503	H	-6.534844	1.175871	0.714263
C	-2.447700	1.100178	-0.180032	H	-6.537779	0.689869	-0.981314
C	-3.928231	1.290269	-0.232213	H	0.476545	-3.744278	-0.578703
C	-4.771221	0.071113	0.110215	H	-1.081279	-4.281229	0.100264
C	-6.261692	0.373376	0.026362	H	-1.052914	-3.027455	-1.167832
(7*R*)-**1**-b							
C	-1.414546	-0.991534	-0.002015	O	-0.127203	2.748141	0.798987
C	-2.375427	-0.058149	0.061539	C	-0.138789	3.653304	-0.309335
C	-2.043423	1.328582	0.339856	H	-1.604665	-2.767465	-1.212539
C	-0.590548	1.508455	0.496387	H	-4.047500	-2.745853	-0.634888
C	0.295629	0.482698	0.404325	H	-1.915585	-3.035707	1.567091
O	-0.123189	-0.796705	0.164472	H	2.071168	1.632455	0.765847
C	-1.957658	-2.377997	-0.255749	H	2.225056	-1.380118	0.227378
N	-3.371598	-2.086956	-0.280106	H	4.327830	0.793330	0.838939
C	-3.673726	-0.751448	-0.175214	H	4.428251	-0.881094	1.380068
O	-2.837727	2.248415	0.446697	H	4.528102	-1.652953	-0.987854
O	-4.777553	-0.267282	-0.272704	H	4.462999	0.012680	-1.532715
O	-1.575019	-3.313166	0.706127	H	6.674375	-1.180766	0.226552
C	1.731652	0.626799	0.553734	H	6.826745	-0.801428	-1.490923
C	2.596584	-0.382060	0.443171	H	6.609875	0.497024	-0.315340
C	4.075145	-0.234759	0.569148	H	-1.144302	3.743969	-0.719386
C	4.805266	-0.630440	-0.718163	H	0.193846	4.614087	0.074032
C	6.316672	-0.523622	-0.568402	H	0.551251	3.302872	-1.080137
(7*R*)-**1**-c							
C	-1.346507	-0.975550	0.049272	O	0.113833	2.706122	0.819615
C	-2.255061	0.010963	0.044150	C	0.208958	3.576615	-0.312060
C	-1.857334	1.383782	0.306522	H	-1.577526	-2.766062	-1.130803
C	-0.404930	1.486127	0.527244	H	-4.039215	-2.597648	-0.675169
C	0.424377	0.410212	0.504209	H	-2.037438	-2.954475	1.634901
O	-0.055608	-0.849254	0.274660	H	2.245406	1.467445	0.920854
C	-1.953303	-2.335787	-0.200437	H	2.250037	-1.563332	0.467685
N	-3.345854	-1.968774	-0.300268	H	4.440857	0.504307	1.125754
C	-3.577335	-0.616117	-0.242095	H	4.412462	-1.154912	1.722367
O	-2.601214	2.349497	0.355339	H	5.978780	-1.015380	-0.119182

O	-4.646009	-0.074967	-0.407442	H	4.623768	-2.019483	-0.596614
O	-1.668939	-3.267682	0.797986	H	3.634861	-0.108027	-1.878754
C	1.857598	0.476894	0.719945	H	4.952449	0.935911	-1.348637
C	2.667256	-0.581600	0.676887	H	5.282267	-0.423707	-2.426583
C	4.144733	-0.517123	0.874795	H	-0.767516	3.706912	-0.777999
C	4.915236	-0.991629	-0.364306	H	0.571381	4.530221	0.062075
C	4.683381	-0.098231	-1.575738	H	0.918760	3.168182	-1.034931
(7*R*)-**1**-d							
C	1.421407	0.968159	-0.266863	O	0.152091	-2.844667	-0.621402
C	2.390741	0.041748	-0.256012	C	0.220413	-3.638524	0.567364
C	2.064754	-1.369015	-0.373110	H	1.745377	2.958613	-1.028496
C	0.609493	-1.576659	-0.461295	H	4.065877	2.810084	-0.076508
C	-0.285191	-0.554517	-0.443316	H	1.423586	3.924320	0.914922
O	0.127170	0.745684	-0.355655	H	-2.065317	-1.748089	-0.578218
C	1.949424	2.372184	-0.128191	H	-2.215489	1.310805	-0.480484
N	3.362200	2.092319	-0.000610	H	-4.442030	0.605729	-1.511089
C	3.687622	0.767206	-0.126922	H	-4.332446	-0.927343	-0.650007
O	2.863826	-2.290686	-0.402957	H	-4.403209	0.324295	1.522719
O	4.808408	0.311314	-0.140998	H	-4.491203	1.840128	0.645927
O	1.369710	2.965863	0.995480	H	-6.665585	1.119943	-0.390278
C	-1.724436	-0.722141	-0.523893	H	-6.580114	-0.401253	0.499088
C	-2.589964	0.292200	-0.535125	H	-6.771311	1.120809	1.372064
C	-4.071415	0.132175	-0.595121	H	1.237575	-3.656271	0.957422
C	-4.770132	0.783948	0.601656	H	-0.464288	-3.239094	1.318712
C	-6.284358	0.649502	0.518151	H	-0.086023	-4.642191	0.285533
(7*R*)-**1**-e							
C	1.630480	0.881563	-0.197674	O	-0.504111	-2.520726	-0.571932
C	2.358880	-0.234357	-0.344373	C	-0.507447	-3.378135	0.573677
C	1.709735	-1.526712	-0.481504	H	2.320373	2.805164	-0.888161
C	0.244675	-1.399063	-0.412826	H	4.626882	2.076074	-0.243966
C	-0.387518	-0.207372	-0.250086	H	2.440675	3.659010	1.132450
O	0.317675	0.959023	-0.147094	H	-2.385828	-0.973827	-0.252096
C	2.475467	2.119451	-0.050560	H	-1.830116	2.028821	0.004347
N	3.793239	1.526995	-0.103460	H	-4.135577	1.887413	0.936862
C	3.792688	0.176876	-0.341605	H	-4.199406	2.015856	-0.794844
O	2.270961	-2.599121	-0.636849	H	-4.553711	-0.463058	-0.972311
O	4.771294	-0.510304	-0.525953	H	-4.489624	-0.590011	0.775801
O	2.162648	2.737196	1.162840	H	-6.491201	0.921570	0.958089
C	-1.828453	-0.049797	-0.183414	H	-6.556585	1.035169	-0.801036
C	-2.436635	1.130358	-0.054232	H	-6.856209	-0.511049	-0.005204
C	-3.914409	1.334921	0.016055	H	-1.074990	-4.262004	0.295456
C	-4.766559	0.076467	-0.045540	H	0.510168	-3.658981	0.844005
C	-6.253677	0.396572	0.030929	H	-0.993796	-2.874311	1.411972
(7*R*)-**1**-f							
C	-1.585514	-0.864149	-0.060830	O	0.699449	2.334301	0.851315
C	-2.255594	0.282694	0.124092	C	0.896340	3.271536	-0.211490
C	-1.547079	1.509638	0.446671	H	-2.314378	-2.435191	-1.348641
C	-0.094233	1.285362	0.513778	H	-4.625430	-1.812414	-0.583607
C	0.478107	0.071539	0.301524	H	-2.519811	-2.809876	1.428375
O	-0.281576	-1.029470	0.019999	H	2.503351	0.689299	0.602591
C	-2.493627	-2.036165	-0.348414	H	1.807987	-2.201767	-0.132194
N	-3.776849	-1.381199	-0.251106	H	4.071024	-2.379413	0.989645
C	-3.703939	-0.024810	-0.050110	H	4.173725	-2.250382	-0.739717

O	-2.056264	2.599820	0.649168	H	4.580072	0.115814	1.157802
O	-4.641545	0.738733	-0.034214	H	5.863999	-0.879057	0.520235
O	-2.321491	-3.106245	0.530087	H	5.639994	1.192538	-0.831846
C	1.906245	-0.176933	0.352995	H	5.278615	-0.229913	-1.810836
C	2.457899	-1.366447	0.109618	H	3.968273	0.788818	-1.222284
C	3.920043	-1.679291	0.160184	H	-0.060923	3.644365	-0.575338
C	4.867839	-0.492726	0.296411	H	1.479436	4.089772	0.202566
C	4.939773	0.367063	-0.961762	H	1.448491	2.797395	-1.026487
(7*R*)-**1**-g							
C	-1.604834	0.858177	0.250984	O	0.716105	-2.418084	0.644423
C	-2.267134	-0.306219	0.306369	C	0.865089	-3.205009	-0.540751
C	-1.545329	-1.559785	0.446648	H	-2.467610	2.695188	0.976150
C	-0.091119	-1.337847	0.481672	H	-4.669615	1.858591	0.126319
C	0.472111	-0.104436	0.397156	H	-2.494483	3.647406	-1.000928
O	-0.298768	1.019138	0.291171	H	2.506369	-0.745541	0.545072
C	-2.517281	2.046224	0.097547	H	1.787792	2.203739	0.138569
N	-3.793627	1.369768	0.026688	H	4.137318	2.293923	-0.566172
C	-3.720860	0.012945	0.209494	H	4.088339	2.294665	1.171076
O	-2.043767	-2.670685	0.528237	H	5.857412	0.827551	0.557063
O	-4.662680	-0.742929	0.286028	H	4.579627	-0.214977	1.127164
O	-2.163267	2.744682	-1.058880	H	5.249905	0.379084	-1.808061
C	1.901598	0.141176	0.413809	H	5.595790	-1.128912	-0.960072
C	2.445604	1.350083	0.270393	H	3.928399	-0.668616	-1.301513
C	3.909338	1.658556	0.296884	H	1.463103	-4.069393	-0.265043
C	4.856660	0.464039	0.316447	H	-0.108001	-3.529229	-0.909401
C	4.907526	-0.284960	-1.011855	H	1.383192	-2.625344	-1.308477

Supplementary Table 4. Atomic coordinates (Å) of conformers with boltzmann distribution > 1% of (7*S*)-**1** obtained at the M062x/def2tzvp level of theory in the IEFPCM model (methanol) (T=298.15 K)

(7*S*)-**1**-a							
C	1.624484	-0.885031	-0.093434	O	-0.482469	2.453586	0.740875
C	2.361227	0.206712	0.158679	C	-0.529946	3.434528	-0.299642
C	1.722398	1.477699	0.455679	H	2.324656	-2.487268	-1.358990
C	0.255380	1.357878	0.427239	H	4.616782	-2.046760	-0.425799
C	-0.386742	0.191685	0.154889	H	2.308737	-2.913446	1.418239
O	0.309779	-0.956198	-0.102810	H	-2.380527	0.942973	0.356899
C	2.463194	-2.116386	-0.341716	H	-1.848786	-1.975711	-0.410268
N	3.780253	-1.559126	-0.144828	H	-4.187275	-1.651022	-1.233646
C	3.792268	-0.204781	0.080726	H	-4.185704	-2.118794	0.439438
O	2.293836	2.525408	0.708981	H	-4.519765	0.274086	1.117212
O	4.779890	0.485684	0.181491	H	-4.523437	0.746818	-0.571368
O	2.153850	-3.183012	0.503101	H	-6.538323	-0.691195	-0.979172
C	-1.829631	0.043365	0.120016	H	-6.534312	-1.175417	0.716910
C	-2.447716	-1.100161	-0.179625	H	-6.857977	0.503351	0.280069
C	-3.928256	-1.290411	-0.231015	H	0.476581	3.744429	-0.579571
C	-4.771216	-0.071034	0.110699	H	-1.053385	3.028101	-1.168080
C	-6.261693	-0.373599	0.028006	H	-1.080763	4.281632	0.100246
(7*S*)-**1**-b							
C	1.414381	0.991989	0.002030	O	0.128064	-2.748041	-0.798987
C	2.375512	0.058856	-0.061495	C	0.139646	-3.653119	0.309404
C	2.043876	-1.327971	-0.339759	H	1.604042	2.767965	1.212561
C	0.591059	-1.508224	-0.496371	H	4.046867	2.747002	0.634908
C	-0.295394	-0.482703	-0.404353	H	1.914790	3.036271	-1.567080
O	0.123078	0.796810	-0.164475	H	-2.070603	-1.632926	-0.766011
C	1.957124	2.378596	0.255763	H	-2.225338	1.379587	-0.227441
N	3.371147	2.087936	0.280093	H	-4.428263	0.879913	-1.380446
C	3.673625	0.752504	0.175226	H	-4.327495	-0.794427	-0.839122
O	2.838423	-2.247608	-0.446495	H	-4.463145	-0.013544	1.532436
O	4.777584	0.268632	0.272698	H	-4.528595	1.652012	0.987372
O	1.574218	3.313669	-0.706099	H	-6.609738	-0.498570	0.314839
C	-1.731372	-0.627184	-0.553843	H	-6.827094	0.799961	1.490249
C	-2.596583	0.381438	-0.443300	H	-6.674592	1.179143	-0.227251
C	-4.075094	0.233753	-0.569411	H	-0.192684	-4.614015	-0.073950
C	-4.805468	0.629398	0.717766	H	1.145093	-3.743504	0.719672
C	-6.316829	0.522180	0.567825	H	-0.550641	-3.302791	1.080034
(7*S*)-**1**-c							
C	-1.347913	0.976711	0.037949	O	0.110022	-2.702937	0.822522
C	-2.256250	-0.009986	0.031382	C	0.210518	-3.575923	-0.306765
C	-1.859178	-1.382275	0.297436	H	-1.575001	2.764440	-1.147016
C	-0.407756	-1.483754	0.525009	H	-4.038328	2.596923	-0.699217
C	0.421361	-0.407660	0.503360	H	-2.044129	2.959042	1.616721
O	-0.057877	0.851125	0.268593	H	2.240398	-1.463085	0.932992
C	-1.953965	2.336285	-0.216967	H	2.246679	1.566126	0.469309
N	-3.346128	1.968870	-0.320810	H	4.402437	1.165228	1.735758
C	-3.577653	0.616319	-0.260513	H	4.434350	-0.497654	1.149585
O	-2.602894	-2.348226	0.344079	H	4.625159	2.014815	-0.588342
O	-4.645759	0.074694	-0.427978	H	5.978689	1.015952	-0.095872

O	-1.673166	3.270529	0.780259	H	4.963258	-0.945177	-1.317816
C	1.853493	-0.473310	0.726593	H	3.646271	0.092188	-1.862176
C	2.663045	0.585292	0.684316	H	5.295873	0.407675	-2.403396
C	4.139420	0.522143	0.890729	H	-0.764097	-3.708611	-0.775912
C	4.916639	0.989036	-0.347108	H	0.922369	-3.168166	-1.028006
C	4.693176	0.086764	-1.553514	H	0.572917	-4.528156	0.070877
(7*S*)-**1**-d							
C	-1.585646	-0.864211	0.060334	O	0.699428	2.334197	-0.851687
C	-2.255720	0.282588	-0.124891	C	0.895956	3.271641	0.211003
C	-1.547172	1.509508	-0.447481	H	-2.314751	-2.435122	1.348170
C	-0.094302	1.285278	-0.514201	H	-4.625643	-1.812504	0.582558
C	0.478029	0.071502	-0.301645	H	-2.519534	-2.810139	-1.428852
O	-0.281683	-1.029498	-0.020169	H	2.503333	0.689303	-0.602220
C	-2.493785	-2.036220	0.347855	H	1.807864	-2.201728	0.132613
N	-3.777006	-1.381307	0.250176	H	4.173433	-2.250243	0.740769
C	-3.704093	-0.024940	0.049041	H	4.071223	-2.379356	-0.988618
O	-2.056348	2.599642	-0.650269	H	5.864017	-0.878918	-0.518748
O	-4.641720	0.738573	0.032857	H	4.580240	0.115877	-1.156743
O	-2.321417	-3.106400	-0.530482	H	3.967710	0.788992	1.223107
C	1.906188	-0.176927	-0.352711	H	5.277888	-0.229685	1.812124
C	2.457814	-1.366404	-0.109086	H	5.639547	1.192725	0.833176
C	3.919983	-1.679203	-0.159230	H	-0.061434	3.644493	0.574494
C	4.867777	-0.492611	-0.295240	H	1.447887	2.797680	1.026253
C	4.939301	0.367243	0.962914	H	1.479137	4.089827	-0.203032
(7*S*)-**1**-f							
C	-1.958929	-0.794425	0.212429	O	1.100322	1.806721	0.606140
C	-2.306758	0.498668	0.271784	C	1.451608	2.508849	-0.591532
C	-1.291943	1.526059	0.409401	H	-3.248992	-2.354786	0.947870
C	0.062237	0.948092	0.446746	H	-5.179335	-0.989047	0.096959
C	0.303666	-0.386195	0.356307	H	-3.510223	-3.287772	-1.011066
O	-0.736146	-1.276220	0.245087	H	1.476558	-2.147851	0.103137
C	-3.143064	-1.714494	0.067676	H	2.887820	0.460034	0.838847
N	-4.207504	-0.737136	0.007063	H	4.581314	-1.299901	1.447244
C	-3.793747	0.557738	0.180552	H	3.792846	-2.449165	0.365691
O	-1.491980	2.727824	0.484875	H	5.833265	-1.596658	-0.653836
O	-4.512329	1.528704	0.253588	H	4.450680	-1.058364	-1.588677
O	-2.991052	-2.480332	-1.090160	H	5.905544	0.666924	0.488727
C	1.576462	-1.092910	0.339550	H	6.169347	0.760288	-1.254154
C	2.787663	-0.586098	0.580068	H	4.610967	1.216367	-0.577080
C	4.040533	-1.394732	0.499650	H	1.786517	1.800696	-1.353395
C	4.967326	-0.931833	-0.633897	H	0.600074	3.081629	-0.958462
C	5.437641	0.510180	-0.485672	H	2.265493	3.179476	-0.328350

Supplementary Table 5. Key transitions, oscillator strengths, and rotatory strengths in the ECD spectrum of conformer (7*R*)-**1**-a at the cam-b3lyp/tzvp level of theory in the IEFPCM model (methanol).

Num	transition	CI-coeff	ΔE (eV)	λ (nm)	f	Rvel	Rlen
1	69->71	0.59629	4.2782	289.80	0.0097	-42.086	-44.0749
2	70->71	0.67615	4.5800	270.71	0.5654	49.6074	50.3474
3	66->72	-0.24556	5.0050	247.72	0.0187	36.1551	38.5485
	69->72	0.4892					
	70->72	0.35973					
4	69->72	-0.34369	5.0708	244.50	0.0334	-17.4234	-17.2612
	70->72	0.5418					
5	68->72	0.55576	5.5147	224.83	0.1763	-38.9947	-41.9624
6	66->72	0.31939	5.6296	220.24	0.1090	41.145	43.4952
	68->71	0.54208					
7	66->72	0.35512	5.6545	219.27	0.0582	-3.6836	-2.2488
	67->72	0.24104					
	68->71	-0.25023					
	68->72	0.23535					
	69->72	0.23356					
8	65->71	0.58188	5.9780	207.40	0.0036	-6.8304	-6.2078
9	66->71	0.32249	6.2159	199.46	0.3722	-43.0099	-42.74
	67->72	0.47693					
10	66->71	0.47424	6.2772	197.52	0.0897	32.594	32.2335
	67->71	0.35324					
11	67->71	0.51421	6.6522	186.38	0.1209	-0.1266	0.5058
	67->72	0.31833					
12	61->72	0.24186	6.7042	184.94	0.0119	-16.3856	-18.9868
	64->72	0.27444					
	65->72	0.41093					
13	61->71	-0.23001	6.9383	178.70	0.0148	3.887	4.2491
	64->71	0.53464					
14	63->71	-0.26206	7.0254	176.48	0.0329	11.3355	12.1151
	65->72	0.43124					
15	63->71	0.45863	7.0995	174.64	0.0201	-29.5875	-30.6506
16	70->73	0.60595	7.1502	173.40	0.0314	25.7979	23.5319
17	70->74	0.6065	7.3517	168.65	0.0035	3.0396	5.6417
	70->76	-0.24288					
18	69->73	0.53641	7.4377	166.70	0.0025	1.6345	2.0597
19	61->72	-0.30233	7.5489	164.24	0.0149	-1.8556	-2.7558
	64->72	0.44516					
	68->73	-0.27917					
20	60->71	0.29479	7.6708	161.63	0.0066	25.3958	27.4012
	61->71	0.26824					
21	60->71	0.22918	7.6780	161.48	0.0074	-3.9609	-4.1554
	70->76	0.31009					
	70->77	0.25532					
	70->80	0.24307					
22	60->71	0.35061	7.7895	159.17	0.0020	-7.4094	-7.933
	61->71	-0.32478					
	62->71	0.26841					
23	68->73	0.44882	7.8426	158.09	0.0966	-45.6346	-45.8038

24	61->71	0.26087	7.8651	157.64	0.0414	-13.2867	-13.4366
	62->71	0.50416					
25	70->76	0.36778	7.9973	155.03	0.0077	-21.864	-25.2683
26	69->74	0.3018	8.0077	154.83	0.0039	9.9585	10.9824
	70->75	0.30684					
27	70->75	0.38649	8.0415	154.18	0.0071	-4.4192	-14.0699
	70->79	0.29833					
28	66->73	0.34629	8.0661	153.71	0.0074	23.5278	25.0565
29	59->71	0.46348	8.1033	153.00	0.0137	26.3526	28.9145
	70->80	-0.33204					
30	68->74	0.26462	8.1539	152.05	0.0173	-35.9217	-32.7982
	68->75	0.29196					
31	59->71	-0.31388	8.2851	149.65	0.0597	47.1271	49.0304
	70->78	0.33235					
	70->80	-0.25206					
32	58->71	-0.25504	8.3243	148.94	0.0292	-32.4458	-34.0569
	58->72	0.3205					
33	58->72	0.22636	8.3971	147.65	0.0256	-4.7431	-3.7734
	67->77	0.22575					
34	69->75	0.23441	8.5306	145.34	0.0218	10.5025	14.6989
35	57->71	0.34212	8.5838	144.44	0.0025	3.295	2.4847
	58->72	-0.25118					
36	67->73	0.4493	8.5999	144.17	0.0684	21.7935	20.804

Number of the excited states; Only transitions with contribution over 10.0% were listed; Configuration-interaction coefficient; Excitation energy; Wavelength; Oscillator strength; Rotatory strength in length form (10-40 cgs); Rotatory strength in velocity form (10-40 cgs).

Supplementary Table 6. Key transitions, oscillator strengths, and rotatory strengths in the ECD spectrum of conformer (7*R*)-**1**-b at the cam-b3lyp/tzvp level of theory in the IEFPCM model (methanol).

Num	transition	CI-coeff	ΔE (eV)	λ (nm)	f	Rvel	Rlen
1	69->71	0.59292	4.2664	290.61	0.0111	-46.0194	-48.009
2	70->71	0.67567	4.5667	271.50	0.6117	56.8668	57.9335
3	66->72	-0.2481	5.0029	247.83	0.0162	34.9458	37.4245
	69->72	0.50351					
	70->72	0.33417					
4	69->72	-0.32155	5.0775	244.19	0.0362	-19.8544	-19.7479
	70->72	0.55709					
5	68->71	0.28672	5.5076	225.12	0.1869	-23.2967	-25.8082
	68->72	0.53693					
6	66->72	0.32052	5.6170	220.73	0.1010	33.4783	35.4054
	68->71	0.52779					
7	66->72	0.35803	5.6465	219.58	0.0570	2.0525	4.5175
	67->72	0.24582					
	68->72	0.25571					
	69->72	0.22857					
8	65->71	0.57473	5.9631	207.92	0.0035	-8.0875	-7.5958
9	66->71	0.36572	6.2087	199.69	0.3444	-58.2071	-57.9422
	67->72	0.45583					
10	66->71	0.43175	6.2604	198.04	0.1256	30.6669	30.9667
	67->71	0.37644					
	67->72	-0.23539					
11	67->71	0.51305	6.6296	187.02	0.1294	-15.2009	-15.8293
	67->72	0.30545					
12	62->72	-0.31124	6.7001	185.05	0.0141	-18.0471	-20.6915
	64->72	-0.27271					
	65->72	0.4087					
13	64->71	0.55355	6.8975	179.75	0.0166	1.4038	1.7237
14	62->72	0.30223	7.0298	176.37	0.0424	7.8231	8.3491
	65->72	0.43339					
	70->73	-0.23304					
15	70->73	0.60798	7.1161	174.23	0.0440	6.3215	4.6564
16	59->71	-0.25766	7.2362	171.34	0.0024	-12.227	-14.9811
	61->71	0.36678					
17	63->71	0.39796	7.3752	168.11	0.0039	8.7876	9.1803
	69->73	-0.36881					
18	70->74	0.57189	7.3993	167.56	0.0013	-0.7115	2.0902
	70->76	-0.28856					
19	63->71	0.34346	7.5275	164.71	0.0021	4.3245	5.4183
	69->73	0.39249					
20	62->72	-0.24503	7.5367	164.51	0.0154	4.1553	3.7748
	64->72	0.46293					
	68->73	-0.28282					
21	62->71	0.39306	7.6807	161.42	0.0081	16.0374	16.177
	62->72	0.26538					
22	70->76	0.2771	7.7085	160.84	0.0079	-6.0318	-5.374
	70->77	0.32981					
	70->78	0.27026					

	70->80	0.26203					
23	64->72	0.25258	7.8308	158.33	0.1344	-68.1892	-69.9666
	68->73	0.50156					
24	66->73	0.32981	8.0029	154.92	0.0019	9.9238	8.5509
	69->74	0.23646					
25	59->71	0.35806	8.0079	154.83	0.0092	-4.01	-4.151
	60->71	-0.30698					
	63->71	0.23493					
26	70->75	0.40768	8.0450	154.11	0.0091	-1.2803	-6.1707
	70->79	0.25176					
	70->80	0.26903					
27	66->73	-0.26065	8.0565	153.89	0.0068	37.4504	36.5547
	69->74	0.33944					
28	58->71	0.26228	8.1002	153.06	0.0268	38.5935	40.9856
	59->71	0.23563					
	60->71	0.24838					
	70->78	0.24019					
	70->80	-0.25777					
29	68->74	0.34195	8.1546	152.04	0.0222	-47.0223	-42.9987
	69->76	-0.23153					
30	70->75	0.34381	8.1743	151.68	0.0014	6.3531	6.9649
	70->76	0.27283					
31	58->71	0.44636	8.2950	149.47	0.0170	28.8526	28.5138
	61->71	-0.28046					
32	59->71	0.24332	8.3200	149.02	0.0329	3.5946	1.6144
	70->78	-0.23432					
33	57->72	0.27781	8.3405	148.65	0.0386	-40.9951	-42.9522
34	57->72	0.32284	8.4072	147.47	0.0215	-1.4228	-0.0387
	68->74	0.2318					
35	68->74	0.27076	8.5452	145.09	0.0292	16.226	21.9749
36	67->73	0.43723	8.5890	144.35	0.0626	18.541	16.107

Number of the excited states; Only transitions with contribution over 10.0% were listed; Configuration-interaction coefficient; Excitation energy; Wavelength; Oscillator strength; Rotatory strength in length form (10-40 cgs); Rotatory strength in velocity form (10-40 cgs).

Supplementary Table 7. Key transitions, oscillator strengths, and rotatory strengths in the ECD spectrum of conformer (7*R*)-**1**-c at the cam-b3lyp/tzvp level of theory in the IEFPCM model (methanol).

Num	transition	CI-coeff	ΔE (eV)	λ (nm)	f	Rvel	Rlen
1	69->71	0.59384	4.2705	290.33	0.0098	-43.0379	-44.8989
2	70->71	0.67691	4.5647	271.62	0.5914	59.8764	61.4015
3	66->72	-0.24734	5.0066	247.64	0.0161	33.8584	36.3229
	69->72	0.49971					
	70->72	0.34021					
4	69->72	-0.32588	5.0769	244.21	0.0349	-21.5136	-21.3822
	70->72	0.55393					
5	68->71	0.29503	5.5085	225.08	0.1836	-11.7734	-13.5395
	68->72	0.53734					
6	66->72	0.32888	5.6187	220.67	0.0970	33.3692	35.0262
	68->71	0.51947					
7	65->71	0.21937	5.6468	219.57	0.0573	-0.7477	1.6303
	66->72	0.35277					
	67->72	0.24469					
	68->71	-0.22174					
	68->72	0.2527					
	69->72	0.226					
8	65->71	0.57409	5.9602	208.02	0.0027	-6.7371	-6.1705
9	66->71	0.35335	6.2106	199.63	0.3471	-61.5153	-61.0668
	67->72	0.4636					
10	66->71	0.44101	6.2633	197.95	0.1124	29.6733	30.1858
	67->71	0.37057					
	67->72	-0.22596					
11	66->71	-0.21881	6.6313	186.97	0.1227	-17.545	-18.4803
	67->71	0.51328					
	67->72	0.30242					
12	62->72	-0.30198	6.6993	185.07	0.0123	-16.1657	-18.611
	64->72	-0.2746					
	65->72	0.40914					
13	64->71	0.54864	6.8973	179.76	0.0194	-2.2702	-2.0459
14	62->72	0.28244	7.0234	176.53	0.0413	8.4383	8.998
	65->72	0.41789					
	70->73	-0.26952					
15	65->72	0.21852	7.0982	174.67	0.0351	0.219	-1.9835
	70->73	0.58653					
16	59->71	-0.29115	7.1539	173.31	0.0030	-15.0176	-18.218
	63->71	0.43564					
17	70->74	0.6291	7.3090	169.63	0.0020	0.42	3.4284
18	61->71	0.41261	7.3794	168.01	0.0040	10.3837	11.0956
	69->73	-0.38103					
19	61->71	0.40634	7.5076	165.15	0.0082	19.8044	21.4985
	69->73	0.33405					
20	62->72	-0.26285	7.5335	164.58	0.0087	-9.0865	-10.0305
	64->72	0.41595					
	68->73	-0.26418					
21	62->71	0.48232	7.6693	161.66	0.0050	3.1959	2.9095
	62->72	0.28083					

22	70->76	0.25238	7.7029	160.96	0.0026	-5.1096	-4.6971
	70->77	0.33063					
	70->78	-0.28755					
23	64->72	0.25745	7.8163	158.62	0.1299	-60.1513	-61.4204
	68->73	0.49283					
24	59->71	0.22702	7.9881	155.21	0.0039	-4.3881	-5.5193
	66->73	-0.22339					
	70->79	0.27053					
25	59->71	0.34466	8.0061	154.86	0.0040	-4.442	-4.6288
	63->71	0.23181					
	70->75	0.26485					
	70->79	-0.2483					
26	59->71	0.23214	8.0180	154.63	0.0095	8.5771	3.5712
	66->73	0.2308					
	69->74	0.25281					
	70->79	0.23868					
27	66->73	-0.27152	8.0444	154.13	0.0051	29.4388	27.4942
	69->74	0.36077					
28	58->71	0.44708	8.0960	153.14	0.0255	38.8852	40.9797
	70->76	-0.23143					
29	58->71	-0.22981	8.1270	152.56	0.0443	55.9453	58.0746
	60->71	0.50241					
30	68->74	0.37097	8.1495	152.14	0.0175	-47.237	-42.3938
	68->75	0.226					
31	70->75	-0.30618	8.2233	150.77	0.0023	-8.9806	-8.7741
	70->76	0.36237					
	70->80	-0.2509					
32	58->71	0.21926	8.3247	148.94	0.0369	-8.335	-10.2589
	60->71	0.21654					
	67->77	0.21788					
33	57->72	0.26735	8.3402	148.66	0.0264	-27.9214	-28.6547
	60->71	0.24586					
	70->78	0.24484					
34	57->71	-0.23324	8.4097	147.43	0.0241	-5.2971	-5.4397
	57->72	0.30046					
35	68->74	-0.23412	8.5205	145.51	0.0372	19.7867	23.8102
	70->80	0.24029					
36	67->73	0.44311	8.5843	144.43	0.0579	27.5271	24.5419

Number of the excited states; Only transitions with contribution over 9.0% were listed; Configuration-interaction coefficient; Excitation energy; Wavelength; Oscillator strength; Rotatory strength in length form (10-40 cgs); Rotatory strength in velocity form (10-40 cgs).

Supplementary Table 8. Key transitions, oscillator strengths, and rotatory strengths in the ECD spectrum of conformer (7*R*)-**1**-d at the cam-b3lyp/tzvp level of theory in the IEFPCM model (methanol).

Num	transition	CI-coeff	ΔE (eV)	λ (nm)	f	Rvel	Rlen
1	69->71	0.60931	4.2827	289.50	0.0057	33.6265	36.3286
2	70->71	0.68155	4.5714	271.22	0.6180	-36.1969	-36.2177
3	66->72	-0.22577	5.0337	246.31	0.0131	-9.3926	-8.716
	69->72	0.50141					
	70->72	0.35143					
4	69->72	-0.30537	5.0991	243.15	0.0376	29.2649	30.2221
	70->72	0.54462					
5	68->71	0.26255	5.4898	225.84	0.1793	7.4591	8.917
	68->72	0.58759					
6	68->71	0.5806	5.6058	221.17	0.1362	-21.797	-23.1181
7	65->71	-0.22637	5.6599	219.06	0.0063	-14.3103	-17.7467
	66->72	0.50187					
	69->72	0.2882					
8	65->71	0.56794	5.9918	206.92	0.0206	35.528	35.9365
9	66->71	0.39661	6.1977	200.05	0.3301	-56.3605	-58.9082
	67->71	-0.23436					
	67->72	0.42277					
10	66->71	0.46038	6.2478	198.45	0.1494	35.4581	34.2406
	67->71	0.28335					
	67->72	-0.29349					
11	67->71	0.54753	6.6068	187.66	0.1377	5.9645	8.3714
	67->72	0.34293					
12	64->71	-0.25543	6.8543	180.88	0.0248	0.0198	-0.3274
	65->72	0.50357					
13	64->71	0.52635	6.9020	179.63	0.0041	0.6375	0.2717
	65->72	0.26498					
14	62->71	-0.2249	7.0660	175.47	0.0129	17.5779	16.4272
	62->72	0.41357					
	64->72	0.31862					
	70->73	0.31463					
15	70->73	0.55542	7.1481	173.45	0.0492	10.8483	11.4894
16	59->71	0.26849	7.2735	170.46	0.0165	-10.8395	-10.4036
	60->71	0.26764					
	61->71	0.41637					
17	63->71	0.38925	7.3929	167.71	0.0077	-17.1743	-17.9521
	69->73	0.37124					
18	70->75	0.45039	7.4653	166.08	0.0001	0.5284	0.0825
	70->76	0.39773					
19	63->71	0.32239	7.5198	164.88	0.0135	21.0538	22.1666
	69->73	-0.23532					
20	64->72	0.36298	7.5853	163.45	0.0016	4.4797	4.4799
	68->73	-0.26772					
	69->73	-0.27712					
21	70->74	-0.2851	7.6935	161.15	0.0017	-13.6975	-13.799
	70->76	0.27689					
	70->77	0.32907					
	70->78	0.23397					

	70->80	-0.27773					
22	68->73	0.43172	7.8289	158.37	0.0796	2.8631	2.1351
23	59->71	0.36254	7.8851	157.24	0.0272	-29.5729	-30.3629
24	62->71	0.43384	7.9072	156.80	0.0263	19.0444	17.402
	62->72	0.2773					
25	57->72	-0.25288	7.9764	155.44	0.0102	-16.4172	-17.7379
	62->71	0.25549					
26	57->72	0.26841	8.0065	154.85	0.0272	13.9543	16.8291
27	67->74	-0.33536	8.0359	154.29	0.0107	-18.1478	-17.2099
	68->74	0.35509					
28	60->71	0.23748	8.0893	153.27	0.0151	-10.0687	-5.0596
	66->73	0.23679					
	69->76	0.25104					
29	69->76	0.32901	8.1058	152.96	0.0064	19.334	19.5146
	69->78	0.22436					
30	70->79	0.34749	8.1431	152.26	0.0053	-9.7776	-10.7607
31	70->75	0.34565	8.1748	151.67	0.0134	9.5654	9.946
	70->76	-0.34311					
32	58->71	0.4177	8.3035	149.32	0.0201	-37.2904	-38.621
	61->71	0.27479					
33	58->71	0.26589	8.3455	148.56	0.0655	-12.0604	-7.8543
	70->78	0.34869					
	70->79	0.25049					
34	67->73	0.3152	8.4904	146.03	0.0611	-16.2419	-18.521
	68->74	-0.28934					
	68->77	0.31447					
35	67->73	0.31905	8.5425	145.14	0.0259	3.3379	3.8804
	69->74	0.2338					
36	70->74	0.41101	8.5908	144.32	0.0116	5.2783	7.9527
	70->77	0.29754					

Number of the excited states; Only transitions with contribution over 10.0% were listed; Configuration-interaction coefficient; Excitation energy; Wavelength; Oscillator strength; Rotatory strength in length form (10-40 cgs); Rotatory strength in velocity form (10-40 cgs).

Supplementary Table 9. Key transitions, oscillator strengths, and rotatory strengths in the ECD spectrum of conformer (7*R*)-**1**-e at the cam-b3lyp/tzvp level of theory in the IEFPCM model (methanol).

Num	transition	CI-coeff	ΔE (eV)	λ (nm)	f	Rvel	Rlen
1	69->71	0.61355	4.2927	288.83	0.0037	26.3185	28.8036
2	70->71	0.68317	4.5856	270.38	0.5758	-27.6931	-27.4851
3	66->72	-0.22774	5.0378	246.11	0.0132	-11.1088	-10.51
	69->72	0.49821					
	70->72	0.3547					
4	69->72	-0.30931	5.0955	243.32	0.0352	27.9349	29.0494
	70->72	0.54446					
5	68->71	0.23399	5.5055	225.20	0.1787	21.3829	23.1072
	68->72	0.59718					
6	68->71	0.58708	5.6251	220.41	0.1400	-23.2417	-24.5274
7	66->72	0.50308	5.6674	218.77	0.0085	-12.9642	-15.7993
	69->72	0.29076					
8	65->71	0.57417	5.9976	206.72	0.0193	32.4241	32.6931
9	66->71	0.38147	6.2115	199.60	0.3389	-70.6368	-72.5015
	67->71	-0.23567					
	67->72	0.43902					
10	66->71	0.47204	6.2576	198.13	0.1279	34.1776	33.1009
	67->71	0.28903					
	67->72	-0.27489					
11	67->71	0.54615	6.6359	186.84	0.1247	-2.5106	-0.4843
	67->72	0.34656					
12	65->72	0.54236	6.8576	180.80	0.0191	-2.7984	-3.4833
13	64->71	0.55335	6.9341	178.80	0.0061	-0.8058	-1.0429
14	61->72	0.36777	7.0688	175.40	0.0072	9.1775	8.2905
	62->72	0.2366					
	64->72	0.34108					
15	63->71	0.53293	7.1171	174.21	0.0055	21.8051	21.2512
16	70->73	0.59473	7.1842	172.58	0.0575	-14.5349	-13.6958
17	70->74	0.25308	7.4015	167.51	0.0006	0.5488	-0.5173
	70->75	0.50687					
	70->76	-0.29175					
18	69->73	0.50712	7.4371	166.71	0.0165	-10.2669	-10.9638
19	61->72	-0.30701	7.5812	163.54	0.0060	4.0851	3.7738
	64->72	0.40805					
	68->73	-0.27479					
20	70->74	0.33601	7.6697	161.65	0.0010	-4.4752	-4.7611
	70->76	0.33415					
	70->77	0.33572					
	70->80	0.27236					
21	57->71	-0.2476	7.7252	160.49	0.0085	28.5222	30.4185
	60->71	0.4605					
22	62->71	0.36132	7.8620	157.70	0.0596	-20.9071	-22.9621
	68->73	0.36157					
23	62->71	0.47987	7.8743	157.45	0.0556	-17.1865	-17.3866
	68->73	-0.28641					
24	59->71	-0.30354	7.9167	156.61	0.0128	-0.0114	-1.5437
	61->71	0.40025					

25	58->71	-0.23511	7.9494	155.97	0.0127	-1.5916	-2.3262
	58->72	0.34961					
26	70->76	0.28594	8.0054	154.88	0.0122	11.6045	14.7665
	70->79	0.32586					
	70->80	-0.26313					
27	61->71	0.34724	8.0183	154.63	0.0097	16.3	18.0711
	66->73	0.26915					
28	67->74	-0.25102	8.0354	154.30	0.0015	-13.953	-14.6851
	68->74	0.31435					
29	67->74	-0.23752	8.0663	153.71	0.0086	-19.7333	-21.2196
	70->75	0.29378					
30	69->75	-0.26091	8.0859	153.33	0.0110	14.3981	24.4119
	69->76	0.30791					
	69->79	-0.25036					
31	59->71	0.34426	8.1480	152.16	0.0095	11.9862	9.9401
	66->73	-0.25508					
	70->78	0.27508					
32	59->71	-0.26196	8.2990	149.40	0.0791	-42.0144	-40.9482
	70->78	0.46891					
33	58->71	0.301	8.4843	146.13	0.0674	-32.6028	-32.7903
	67->73	0.25489					
34	68->74	0.23518	8.5040	145.79	0.0109	-14.6735	-17.9598
	68->77	0.30303					
35	70->74	0.36644	8.5563	144.90	0.0080	8.6491	10.4302
	70->77	-0.28799					
36	67->73	0.33305	8.5888	144.36	0.0265	-10.8592	-11.5079
	70->74	0.30854					
	70->77	-0.2369					

Number of the excited states; Only transitions with contribution over 10.0% were listed; Configuration-interaction coefficient; Excitation energy; Wavelength; Oscillator strength; Rotatory strength in length form (10-40 cgs); Rotatory strength in velocity form (10-40 cgs).

Supplementary Table 10. Key transitions, oscillator strengths, and rotatory strengths in the ECD spectrum of conformer (7*R*)-**1**-f at the cam-b3lyp/tzvp level of theory in the IEFPCM model (methanol).

Num	transition	CI-coeff	ΔE (eV)	λ (nm)	f	Rvel	Rlen
1	69->71	0.59747	4.2807	289.64	0.0079	-37.8142	-39.6086
2	70->71	0.679	4.5676	271.44	0.5554	45.5945	46.6666
3	66->72	-0.24978	5.0078	247.58	0.0171	35.0813	37.6492
	69->72	0.49813					
	70->72	0.34412					
4	69->72	-0.32983	5.0672	244.68	0.0332	-18.4057	-18.2167
	70->72	0.55191					
5	68->71	0.22772	5.5088	225.07	0.1756	-28.8991	-30.9572
	68->72	0.55939					
6	66->72	0.32138	5.6265	220.36	0.1044	36.4573	38.1561
	68->71	0.53684					
7	65->71	-0.21969	5.6495	219.46	0.0546	-6.8747	-5.6175
	66->72	0.36009					
	67->72	0.23358					
	68->71	-0.25088					
	68->72	0.22273					
	69->72	0.23109					
8	65->71	0.57775	5.9624	207.94	0.0019	-4.5309	-3.7517
9	66->71	0.3033	6.2116	199.60	0.3750	-46.7974	-46.3472
	67->71	-0.21841					
	67->72	0.48574					
10	66->71	0.48811	6.2729	197.65	0.0723	31.3716	31.1921
	67->71	0.33973					
11	67->71	0.51229	6.6439	186.61	0.1099	0.8053	1.1561
	67->72	0.30759					
12	61->72	0.28141	6.6993	185.07	0.0085	-12.518	-14.5619
	64->72	0.27827					
	65->72	0.40574					
13	64->71	0.55759	6.9154	179.29	0.0248	-2.3105	-2.5283
14	61->72	-0.21389	7.0024	177.06	0.0218	7.3264	7.9859
	63->71	0.36739					
	65->72	0.35708					
	70->73	0.22866					
15	61->72	0.22539	7.0581	175.66	0.0244	-23.8269	-26.2563
	63->71	0.41186					
	64->72	0.22193					
	65->72	-0.24316					
16	70->73	0.61717	7.1114	174.35	0.0287	5.7209	4.971
17	70->74	0.63578	7.3171	169.44	0.0003	0.1112	0.7233
18	62->71	-0.31362	7.3966	167.62	0.0042	7.254	7.5347
	69->73	0.44245					
19	62->71	-0.25086	7.5262	164.74	0.0155	10.2678	9.9935
	64->72	0.40799					
	68->73	-0.25406					
20	61->72	-0.21698	7.5354	164.54	0.0017	-8.1581	-8.6306
	62->71	0.38793					
	69->73	0.32063					

21	70->75	0.2666	7.6736	161.57	0.0003	0.4476	0.2811
	70->76	0.33553					
	70->77	-0.28479					
	70->78	-0.21687					
	70->80	-0.26318					
22	61->71	0.50308	7.7274	160.45	0.0152	-0.0068	0.5188
	61->72	0.28164					
23	64->72	0.21836	7.8198	158.55	0.0945	-34.1277	-33.5601
	68->73	0.46758					
24	59->71	-0.23571	7.9749	155.47	0.0440	-43.3579	-49.4445
	70->76	0.344					
	70->79	0.26112					
25	69->74	0.37281	8.0098	154.79	0.0141	-3.9669	-10.2037
26	66->73	0.30541	8.0466	154.08	0.0106	38.1471	39.8552
	69->74	-0.24041					
27	59->71	0.24122	8.0606	153.81	0.0045	3.9286	6.1382
	70->75	0.41447					
	70->76	-0.21685					
	70->79	0.23548					
28	59->71	0.27345	8.0932	153.20	0.0115	-10.8827	-10.3326
	60->71	0.49451					
29	59->71	0.27958	8.0981	153.10	0.0054	18.4004	20.4277
	70->78	-0.28006					
	70->80	0.31746					
30	68->74	0.25998	8.1547	152.04	0.0180	-42.9159	-40.6388
	68->75	0.27802					
	69->75	-0.22277					
31	59->71	0.29698	8.2401	150.46	0.0529	55.7808	59.4724
	70->78	0.33319					
32	57->72	0.2635	8.3220	148.98	0.0366	-32.0081	-34.2557
	67->77	0.21907					
33	67->77	0.21651	8.3966	147.66	0.0192	-4.779	-4.3447
34	58->71	0.37846	8.4935	145.97	0.0080	4.2626	5.0972
35	68->74	0.21594	8.5567	144.90	0.0202	23.6873	26.5386
	69->75	0.29511					
36	67->73	0.44389	8.5922	144.30	0.0680	28.9755	27.9453

Number of the excited states; Only transitions with contribution over 9.0% were listed; Configuration-interaction coefficient; Excitation energy; Wavelength; Oscillator strength; Rotatory strength in length form (10-40 cgs); Rotatory strength in velocity form (10-40 cgs).

Supplementary Table 11. Key transitions, oscillator strengths, and rotatory strengths in the ECD spectrum of conformer (7*R*)-**1**-g at the cam-b3lyp/tzvp level of theory in the IEFPCM model (methanol).

Num	transition	CI-coeff	ΔE (eV)	λ (nm)	f	Rvel	Rlen
1	69->71	0.61088	4.2953	288.65	0.0034	25.2774	27.6986
2	70->71	0.68402	4.5778	270.84	0.5636	-25.7718	-25.9549
3	66->72	-0.23887	5.0400	246.00	0.0109	-7.1504	-6.4831
	69->72	0.51931					
	70->72	0.31706					
4	69->72	-0.27375	5.0907	243.55	0.0351	24.7579	25.4034
	70->72	0.56718					
5	68->71	0.24113	5.5000	225.43	0.1738	12.202	13.4
	68->72	0.59479					
6	68->71	0.57814	5.6208	220.58	0.1356	-26.478	-27.9433
7	65->71	-0.23134	5.6630	218.94	0.0078	-8.008	-10.4168
	66->72	0.49499					
	69->72	0.28207					
8	65->71	0.57255	5.9764	207.45	0.0189	32.996	33.3401
9	66->71	0.3799	6.2015	199.93	0.3248	-66.9605	-69.2062
	67->71	-0.23522					
	67->72	0.44565					
10	66->71	0.4785	6.2555	198.20	0.1249	38.115	37.1409
	67->71	0.28387					
	67->72	-0.26742					
11	67->71	0.54922	6.6283	187.05	0.1161	2.0783	4.6178
	67->72	0.34023					
12	65->72	0.55916	6.8426	181.19	0.0170	-2.8593	-3.6083
13	64->71	0.58029	6.9216	179.13	0.0119	-0.5994	-0.5615
14	63->71	0.52397	7.0424	176.05	0.0008	-0.2423	0.135
15	61->71	-0.23561	7.0776	175.18	0.0124	31.117	29.7719
	61->72	0.35568					
	63->71	-0.2667					
	64->72	0.27364					
	70->73	-0.22728					
16	70->73	0.58457	7.1494	173.42	0.0434	-0.6709	-0.1829
17	70->74	0.32732	7.3746	168.12	0.0011	0.1729	0.7491
	70->75	0.53294					
18	62->71	-0.30122	7.4038	167.46	0.0104	-17.293	-18.1906
	69->73	0.44166					
19	62->71	0.38217	7.5236	164.79	0.0188	20.1793	21.6307
	64->72	-0.30645					
20	62->71	0.30391	7.5825	163.51	0.0007	4.53	5.4134
	64->72	0.30904					
	68->73	0.24321					
	69->73	0.29					
21	70->74	0.29671	7.6479	162.12	0.0001	1.8505	2.0156
	70->76	0.42422					
	70->80	-0.25492					
22	68->73	0.47678	7.8476	157.99	0.0949	-4.732	-6.4219
23	59->71	-0.31976	7.9120	156.70	0.0095	-2.8839	-4.3876
	61->71	0.39465					

	66->73	0.23115					
24	58->72	0.29848	7.9332	156.29	0.0143	6.7055	5.7867
	59->71	0.27515					
25	61->71	0.32099	7.9913	155.15	0.0178	-12.7755	-14.0558
26	58->72	-0.25332	8.0132	154.72	0.0220	29.8623	34.9428
	61->71	0.26374					
	70->79	-0.24015					
27	67->74	-0.27915	8.0357	154.29	0.0051	-13.8771	-11.95
	68->74	0.31143					
28	69->75	0.33836	8.0747	153.55	0.0118	6.1833	10.1826
	69->79	0.23863					
29	60->71	0.32716	8.0882	153.29	0.0213	-35.9605	-36.4437
	70->77	-0.31883					
30	60->71	0.41596	8.1286	152.53	0.0036	9.518	8.2525
	66->73	0.22789					
31	70->75	0.2702	8.1445	152.23	0.0042	7.3211	6.1081
	70->76	0.2997					
	70->78	0.22927					
32	59->71	0.25041	8.2431	150.41	0.0671	-26.3442	-25.443
	70->78	0.30914					
33	57->71	0.27806	8.4752	146.29	0.0612	-45.6925	-44.5978
	67->73	0.24091					
34	57->71	0.23501	8.4881	146.07	0.0095	-5.886	-8.0467
	68->77	0.23464					
35	67->73	0.31105	8.5497	145.02	0.0340	-0.9692	-1.287
	70->74	0.23024					
36	57->71	0.22529	8.5810	144.49	0.0073	-17.2137	-17.4458
	58->71	0.25386					
	70->74	0.30758					

Number of the excited states; Only transitions with contribution over 10.0% were listed; Configuration-interaction coefficient; Excitation energy; Wavelength; Oscillator strength; Rotatory strength in length form (10-40 cgs); Rotatory strength in velocity form (10-40 cgs).

Supplementary Table 12. Key transitions, oscillator strengths, and rotatory strengths in the ECD spectrum of conformer (7*S*)-**1**-a at the cam-b3lyp/tzvp level of theory in the IEFPCM model (methanol).

Num	transition	CI-coeff	ΔE (eV)	λ (nm)	f	Rvel	Rlen
1	69->71	0.59631	4.2782	289.80	0.0097	42.0578	44.0473
2	70->71	0.67616	4.5800	270.71	0.5654	-49.5892	-50.3281
3	66->72	-0.24561	5.0050	247.72	0.0188	-36.1476	-38.5387
	69->72	0.48902					
	70->72	0.36					
4	69->72	-0.34399	5.0709	244.50	0.0333	17.4204	17.2592
	70->72	0.54157					
5	68->72	0.55595	5.5144	224.84	0.1760	39.0538	42.0263
6	66->72	0.31738	5.6295	220.24	0.1097	-40.8576	-43.1911
	68->71	0.54346					
7	66->72	0.35714	5.6546	219.26	0.0577	3.365	1.9103
	67->72	0.23986					
	68->71	-0.24795					
	68->72	0.23501					
	69->72	0.23423					
8	65->71	0.5819	5.9781	207.40	0.0036	6.8461	6.2202
9	66->71	0.32223	6.2158	199.47	0.3723	43.0136	42.7466
	67->72	0.47702					
10	66->71	0.47493	6.2773	197.51	0.0897	-32.6214	-32.2605
	67->71	0.35241					
11	67->71	0.51458	6.6520	186.39	0.1209	0.0157	-0.623
	67->72	0.3186					
12	61->72	-0.24197	6.7044	184.93	0.0121	16.4732	19.0829
	64->72	-0.27408					
	65->72	0.41122					
13	61->71	-0.22983	6.9383	178.70	0.0147	-3.8768	-4.2395
	64->71	0.53462					
14	63->71	-0.26251	7.0256	176.48	0.0329	-11.3543	-12.1349
	65->72	0.43111					
15	63->71	0.45838	7.0997	174.63	0.0201	29.533	30.6032
16	70->73	0.6063	7.1505	173.39	0.0315	-25.7125	-23.4538
17	70->74	0.60649	7.3517	168.65	0.0034	-3.0266	-5.63
	70->76	-0.2429					
18	69->73	0.53636	7.4379	166.69	0.0025	-1.6348	-2.0606
19	61->72	-0.30225	7.5489	164.24	0.0149	1.8483	2.7465
	64->72	0.44542					
	68->73	0.279					
20	60->71	0.28756	7.6709	161.63	0.0062	-24.7178	-26.6644
	61->71	0.26429					
21	60->71	-0.2387	7.6777	161.49	0.0078	3.2382	3.3656
	70->76	0.30303					
	70->77	0.2506					
	70->80	0.23798					
22	60->71	0.35024	7.7897	159.17	0.0020	7.416	7.9428
	61->71	-0.32519					
	62->71	-0.26852					
23	68->73	0.4485	7.8427	158.09	0.0965	45.4015	45.5672

24	61->71	-0.26061	7.8652	157.64	0.0415	13.3116	13.4633
	62->71	0.50411					
25	70->76	0.36926	7.9971	155.04	0.0079	22.1517	25.5613
26	69->74	0.3035	8.0077	154.83	0.0038	-10.1321	-11.1177
	70->75	0.30464					
27	70->75	0.38765	8.0415	154.18	0.0070	4.4796	14.0819
	70->79	0.29855					
28	66->73	0.34686	8.0663	153.71	0.0074	-23.555	-25.0742
29	59->71	0.46384	8.1034	153.00	0.0137	-26.2808	-28.8408
	70->80	-0.33169					
30	68->74	0.26493	8.1537	152.06	0.0174	35.9985	32.8905
	68->75	0.29195					
31	59->71	-0.31347	8.2852	149.64	0.0596	-47.1462	-49.0528
	70->78	0.33253					
	70->80	-0.25251					
32	58->71	-0.25512	8.3242	148.94	0.0292	32.3784	33.9832
	58->72	0.32044					
33	58->72	0.22649	8.3970	147.65	0.0256	4.7363	3.7751
	67->77	0.2259					
34	69->75	0.23483	8.5306	145.34	0.0217	-10.5371	-14.7275
35	57->71	0.34222	8.5838	144.44	0.0026	-3.259	-2.4458
	58->72	-0.25106					
36	67->73	0.44976	8.5998	144.17	0.0683	-21.7149	-20.7367

Number of the excited states; Only transitions with contribution over 10.0% were listed; Configuration-interaction coefficient; Excitation energy; Wavelength; Oscillator strength; Rotatory strength in length form (10-40 cgs); Rotatory strength in velocity form (10-40 cgs).

Supplementary Table 13. Key transitions, oscillator strengths, and rotatory strengths in the ECD spectrum of conformer (7*S*)-**1**-b at the cam-b3lyp/tzvp level of theory in the IEFPCM model (methanol).

Num	transition	CI-coeff	ΔE (eV)	λ (nm)	f	Rvel	Rlen
1	69->71	0.59292	4.2664	290.60	0.0111	45.9786	47.966
2	70->71	0.67569	4.5667	271.50	0.6117	-56.8115	-57.8779
3	66->72	-0.24818	5.0029	247.83	0.0162	-34.9397	-37.4185
	69->72	0.50373					
	70->72	0.33377					
4	69->72	-0.32122	5.0774	244.19	0.0362	19.8418	19.7377
	70->72	0.55733					
5	68->71	0.28672	5.5076	225.12	0.1869	23.3058	25.814
	68->72	0.53692					
6	66->72	0.32068	5.6170	220.73	0.1010	-33.5026	-35.4314
	68->71	0.52769					
7	66->72	0.35785	5.6464	219.58	0.0570	-2.0153	-4.4784
	67->72	0.24592					
	68->72	0.25576					
	69->72	0.22846					
8	65->71	0.57474	5.9630	207.92	0.0035	8.0724	7.5792
9	66->71	0.36575	6.2087	199.69	0.3445	58.2158	57.9537
	67->72	0.45582					
10	66->71	0.4317	6.2604	198.04	0.1256	-30.6787	-30.9767
	67->71	0.3765					
	67->72	-0.23541					
11	67->71	0.51303	6.6296	187.02	0.1293	15.1981	15.8227
	67->72	0.30543					
12	62->72	-0.31123	6.7001	185.05	0.0141	18.0469	20.6899
	64->72	0.27261					
	65->72	0.4088					
13	64->71	0.55359	6.8975	179.75	0.0166	-1.4032	-1.7226
14	62->72	0.30235	7.0297	176.37	0.0423	-7.8376	-8.3636
	65->72	0.43346					
	70->73	0.23261					
15	70->73	0.60815	7.1161	174.23	0.0441	-6.3002	-4.6328
16	59->71	0.25764	7.2362	171.34	0.0024	12.2188	14.9707
	61->71	0.3668					
17	63->71	0.39799	7.3752	168.11	0.0039	-8.7866	-9.1785
	69->73	-0.36891					
18	70->74	0.57202	7.3993	167.56	0.0013	0.7138	-2.0865
	70->76	-0.28858					
19	63->71	0.34354	7.5274	164.71	0.0021	-4.3508	-5.445
	69->73	0.39245					
20	62->72	0.2451	7.5367	164.51	0.0154	-4.1381	-3.7556
	64->72	0.46298					
	68->73	-0.28281					
21	62->71	0.3932	7.6807	161.42	0.0082	-16.0397	-16.1802
	62->72	0.26541					
22	70->76	0.2772	7.7085	160.84	0.0079	6.0269	5.3717
	70->77	0.32991					
	70->78	0.27029					

	70->80	0.26211					
23	64->72	0.25258	7.8308	158.33	0.1345	68.1829	69.9623
	68->73	0.50159					
24	66->73	0.32989	8.0028	154.93	0.0019	-9.9203	-8.5395
	69->74	0.23646					
25	59->71	0.35809	8.0079	154.83	0.0092	4.0029	4.1448
	60->71	-0.307					
	63->71	0.23496					
26	70->75	0.40754	8.0450	154.11	0.0091	1.2945	6.1978
	70->79	0.25184					
	70->80	0.26883					
27	66->73	-0.26068	8.0565	153.89	0.0068	-37.487	-36.5974
	69->74	0.33923					
28	58->71	0.26223	8.1002	153.06	0.0268	-38.5742	-40.9696
	59->71	0.23574					
	60->71	0.24836					
	70->78	0.24019					
	70->80	-0.25791					
29	68->74	0.34202	8.1546	152.04	0.0222	47.0279	43.0023
	69->76	-0.23161					
30	70->75	0.34392	8.1743	151.67	0.0014	-6.3595	-6.9691
	70->76	0.27294					
31	58->71	0.44642	8.2951	149.47	0.0170	-28.8558	-28.5152
	61->71	0.28041					
32	59->71	0.2433	8.3200	149.02	0.0328	-3.5681	-1.5877
	70->78	-0.23417					
33	57->72	0.27782	8.3404	148.65	0.0386	40.9883	42.9444
34	57->72	0.32275	8.4071	147.47	0.0215	1.4275	0.0433
	68->74	-0.23182					
35	68->74	0.2708	8.5452	145.09	0.0293	-16.2358	-21.9853
36	67->73	0.43719	8.5891	144.35	0.0626	-18.5349	-16.1017

Number of the excited states; Only transitions with contribution over 10.0% were listed; Configuration-interaction coefficient; Excitation energy; Wavelength; Oscillator strength; Rotatory strength in length form (10-40 cgs); Rotatory strength in velocity form (10-40 cgs).

Supplementary Table 14. Key transitions, oscillator strengths, and rotatory strengths in the ECD spectrum of conformer (7*S*)-**1**-c at the cam-b3lyp/tzvp level of theory in the IEFPCM model (methanol).

Num	transition	CI-coeff	ΔE (eV)	λ (nm)	f	Rvel	Rlen
1	69->71	0.59387	4.2703	290.34	0.0098	42.9682	44.824
2	70->71	0.67692	4.5647	271.61	0.5914	-59.8747	-61.3931
3	66->72	-0.24754	5.0065	247.65	0.0160	-33.8467	-36.3037
	69->72	0.50048					
	70->72	0.33899					
4	69->72	-0.32482	5.0768	244.22	0.0350	21.4585	21.3324
	70->72	0.55466					
5	68->71	0.29457	5.5084	225.08	0.1835	11.8635	13.6404
	68->72	0.5374					
6	66->72	0.32956	5.6185	220.67	0.0968	-33.6019	-35.2778
	68->71	0.51921					
7	65->71	-0.21916	5.6467	219.57	0.0576	1.0102	-1.3599
	66->72	0.35207					
	67->72	0.24495					
	68->71	-0.22276					
	68->72	0.25282					
	69->72	0.22561					
8	65->71	0.57419	5.9603	208.02	0.0028	6.7901	6.229
9	66->71	0.35339	6.2105	199.64	0.3471	61.5372	61.0938
	67->72	0.46356					
10	66->71	0.44118	6.2633	197.95	0.1125	-29.6856	-30.1969
	67->71	0.37054					
	67->72	-0.22598					
11	66->71	-0.21886	6.6312	186.97	0.1227	17.5239	18.4548
	67->71	0.51337					
	67->72	0.30248					
12	62->72	-0.30165	6.6995	185.07	0.0123	16.1677	18.6147
	64->72	0.27462					
	65->72	0.40928					
13	64->71	0.54853	6.8973	179.76	0.0193	2.3542	2.1323
14	62->72	0.28244	7.0235	176.53	0.0414	-8.3879	-8.9437
	65->72	0.41782					
	70->73	0.2694					
15	65->72	-0.21838	7.0980	174.67	0.0349	-0.1076	2.1004
	70->73	0.58635					
16	59->71	-0.29128	7.1539	173.31	0.0030	14.8912	18.1034
	63->71	0.4357					
17	70->74	0.62909	7.3093	169.63	0.0020	-0.4732	-3.4912
18	61->71	0.41317	7.3794	168.01	0.0040	-10.3299	-11.0463
	69->73	-0.38085					
19	61->71	0.40674	7.5074	165.15	0.0081	-19.6879	-21.3693
	69->73	0.33533					
20	62->72	0.2621	7.5335	164.58	0.0088	8.8836	9.8249
	64->72	0.41709					
	68->73	-0.265					
21	62->71	0.48278	7.6697	161.65	0.0049	-3.1548	-2.864
	62->72	0.28055					

22	70->76	0.25145	7.7031	160.95	0.0026	5.0363	4.6369
	70->77	0.33067					
	70->78	-0.28693					
23	64->72	0.2575	7.8164	158.62	0.1300	60.3286	61.6066
	68->73	0.49284					
24	59->71	0.22964	7.9883	155.21	0.0038	4.0965	5.2358
	66->73	-0.22544					
	70->79	0.26264					
25	59->71	0.34632	8.0063	154.86	0.0037	3.9964	4.0801
	63->71	0.23064					
	70->75	0.26499					
	70->79	-0.24816					
26	59->71	0.22843	8.0180	154.63	0.0097	-8.0827	-2.9626
	66->73	0.22508					
	69->74	0.25238					
	70->79	0.24805					
27	66->73	-0.27299	8.0443	154.13	0.0051	-29.4305	-27.519
	69->74	0.35943					
28	58->71	0.44945	8.0960	153.14	0.0250	-38.5415	-40.5975
	63->71	-0.21277					
	70->76	-0.22977					
29	58->71	-0.22367	8.1261	152.57	0.0449	-56.0823	-58.2237
	60->71	0.50479					
30	68->74	0.3711	8.1493	152.14	0.0176	47.1346	42.2948
	68->75	0.22623					
31	70->75	-0.30611	8.2236	150.77	0.0023	8.9486	8.729
	70->76	0.36292					
	70->80	-0.25012					
32	58->71	0.22088	8.3243	148.94	0.0370	8.1828	10.1043
	60->71	0.21762					
	67->77	0.21736					
33	57->72	0.26834	8.3398	148.66	0.0263	28.2163	28.9608
	60->71	-0.24582					
	70->78	-0.24494					
34	57->71	-0.23289	8.4094	147.44	0.0240	5.4423	5.5927
	57->72	0.29957					
35	68->74	-0.23424	8.5205	145.51	0.0374	-20.1378	-24.1682
	70->80	0.24308					
36	67->73	0.44328	8.5842	144.43	0.0578	-27.5951	-24.6309

Number of the excited states; Only transitions with contribution over 9.0% were listed; Configuration-interaction coefficient; Excitation energy; Wavelength; Oscillator strength; Rotatory strength in length form (10-40 cgs); Rotatory strength in velocity form (10-40 cgs).

Supplementary Table 15. Key transitions, oscillator strengths, and rotatory strengths in the ECD spectrum of conformer (7*S*)-**1**-d at the cam-b3lyp/tzvp level of theory in the IEFPCM model (methanol).

Num	transition	CI-coeff	ΔE (eV)	λ (nm)	f	Rvel	Rlen
1	69->71	0.59747	4.2807	289.64	0.0079	37.834	39.6288
2	70->71	0.679	4.5676	271.44	0.5554	-45.6141	-46.6872
3	66->72	-0.24973	5.0078	247.58	0.0171	-35.0868	-37.6543
	69->72	0.498					
	70->72	0.34434					
4	69->72	-0.33002	5.0672	244.68	0.0332	18.4128	18.2234
	70->72	0.55178					
5	68->71	0.22774	5.5088	225.07	0.1756	28.894	30.9517
	68->72	0.55939					
6	66->72	0.32139	5.6265	220.36	0.1044	-36.4548	-38.1536
	68->71	0.53684					
7	65->71	-0.21965	5.6495	219.46	0.0546	6.8682	5.6107
	66->72	0.36009					
	67->72	0.2336					
	68->71	-0.25088					
	68->72	0.22272					
	69->72	0.23113					
8	65->71	0.57775	5.9624	207.94	0.0020	4.537	3.758
9	66->71	0.3033	6.2115	199.60	0.3750	46.7936	46.3428
	67->71	-0.21841					
	67->72	0.48574					
10	66->71	0.4881	6.2729	197.65	0.0722	-31.367	-31.1876
	67->71	0.33975					
11	67->71	0.51228	6.6439	186.61	0.1099	-0.8066	-1.1576
	67->72	0.30759					
12	61->72	-0.28142	6.6993	185.07	0.0085	12.5155	14.5594
	64->72	-0.2783					
	65->72	0.4057					
13	64->71	0.55757	6.9154	179.29	0.0248	2.3088	2.5264
14	61->72	0.21384	7.0024	177.06	0.0218	-7.3277	-7.9867
	63->71	0.3674					
	65->72	0.35706					
	70->73	0.22874					
15	61->72	-0.22537	7.0580	175.66	0.0245	23.8361	26.2648
	63->71	0.41185					
	64->72	-0.22193					
	65->72	-0.24317					
16	70->73	0.61713	7.1113	174.35	0.0287	-5.7266	-4.9767
17	70->74	0.63578	7.3171	169.44	0.0003	-0.1111	-0.7232
18	62->71	-0.31363	7.3966	167.62	0.0042	-7.2547	-7.5355
	69->73	0.44245					
19	62->71	0.2507	7.5262	164.74	0.0155	-10.2598	-9.9847
	64->72	0.40805					
	68->73	0.2541					
20	61->72	0.21692	7.5354	164.54	0.0017	8.1516	8.6236
	62->71	0.38802					
	69->73	0.32066					

21	70->75	0.26664	7.6735	161.57	0.0003	-0.4453	-0.2798
	70->76	0.3355					
	70->77	-0.2848					
	70->78	-0.21691					
	70->80	-0.26318					
22	61->71	0.50309	7.7274	160.45	0.0152	0.0034	-0.5223
	61->72	0.28166					
23	64->72	-0.21835	7.8198	158.55	0.0945	34.1168	33.5491
	68->73	0.46756					
24	59->71	-0.23569	7.9748	155.47	0.0440	43.3702	49.4582
	70->76	0.344					
	70->79	0.26115					
25	69->74	0.37278	8.0098	154.79	0.0141	3.9583	10.1934
26	66->73	0.3053	8.0466	154.08	0.0106	-38.1444	-39.8559
	69->74	-0.24039					
27	59->71	0.24118	8.0606	153.82	0.0045	-3.9245	-6.1294
	70->75	0.41437					
	70->76	-0.21688					
	70->79	0.23541					
28	59->71	0.27288	8.0932	153.20	0.0115	10.8813	10.3356
	60->71	0.49495					
29	59->71	0.2802	8.0981	153.10	0.0054	-18.404	-20.4358
	70->78	-0.28004					
	70->80	0.31742					
30	68->74	0.25999	8.1547	152.04	0.0180	42.9202	40.6424
	68->75	0.27802					
	69->75	-0.22278					
31	59->71	0.29698	8.2401	150.46	0.0528	-55.7753	-59.4667
	70->78	0.3332					
32	57->72	0.26354	8.3219	148.98	0.0366	32.006	34.2535
	67->77	0.21905					
33	67->77	0.21654	8.3966	147.66	0.0192	4.783	4.3485
34	58->71	0.37848	8.4935	145.97	0.0080	-4.2605	-5.0952
35	68->74	0.21595	8.5567	144.90	0.0202	-23.688	-26.54
	69->75	0.29512					
36	67->73	0.44387	8.5922	144.30	0.0680	-28.9803	-27.9484

Number of the excited states; Only transitions with contribution over 9.0% were listed; Configuration-interaction coefficient; Excitation energy; Wavelength; Oscillator strength; Rotatory strength in length form (10-40 cgs); Rotatory strength in velocity form (10-40 cgs).

Supplementary Table 16. Key transitions, oscillator strengths, and rotatory strengths in the ECD spectrum of conformer (7*S*)-**1**-e at the cam-b3lyp/tzvp level of theory in the IEFPCM model (methanol).

Num	transition	CI-coeff	ΔE (eV)	λ (nm)	f	Rvel	Rlen
1	69->71	0.5821	4.3312	286.26	0.0027	-14.1613	-16.1607
2	70->71	0.68405	4.6153	268.64	0.4890	19.1947	20.4431
3	66->72	0.23612	5.0033	247.80	0.0116	-21.3542	-23.1176
	69->72	0.56531					
4	70->72	0.66006	5.0767	244.22	0.0437	-1.4651	-1.5167
5	68->71	-0.31051	5.4394	227.94	0.1932	-32.1272	-33.0418
	68->72	0.54444					
6	65->71	-0.29649	5.5503	223.38	0.0550	64.8879	67.1418
	66->72	0.4565					
7	67->72	-0.23284	5.7021	217.44	0.1925	-3.5465	-3.8963
	68->71	0.57458					
	68->72	0.24382					
8	65->71	0.56694	5.8784	210.92	0.0136	-36.9785	-36.9781
	66->72	0.23002					
	69->72	-0.23469					
9	66->71	-0.27174	6.2012	199.94	0.2608	39.7889	38.0816
	67->72	0.5595					
10	66->71	0.49492	6.3070	196.58	0.0648	-25.7801	-24.4034
	66->72	0.25869					
	67->72	0.23494					
11	67->71	0.63791	6.6305	186.99	0.0340	-1.9717	-5.5067
12	65->72	0.59054	6.7577	183.47	0.0037	-5.609	-5.1103
13	64->71	0.50255	6.8401	181.26	0.0010	0.2507	1.2761
	70->73	-0.34845					
14	64->71	0.22881	6.9972	177.19	0.1062	46.9402	47.4114
	70->73	0.50612					
15	61->72	0.26219	7.1081	174.43	0.0008	2.3463	3.1135
	62->72	0.34432					
	63->71	0.25665					
	64->72	0.2747					
16	63->71	0.37456	7.1471	173.47	0.0171	1.2379	2.7181
	64->71	-0.23256					
17	69->73	0.4636	7.3579	168.50	0.0018	-0.3239	-0.8263
18	70->75	0.48093	7.3688	168.25	0.0068	-2.5404	-1.5433
19	61->71	0.34847	7.5176	164.92	0.0097	-8.8908	-8.4596
	62->71	-0.3296					
	63->71	-0.33498					
20	61->72	-0.24107	7.5298	164.66	0.0300	-0.5552	0.3867
	64->72	0.40318					
	68->73	0.31867					
21	64->72	-0.2791	7.7611	159.75	0.0838	19.7467	19.6549
	68->73	0.4958					
22	70->74	0.26252	7.8743	157.45	0.0111	-7.6036	-6.0019
	70->77	0.32954					
	70->81	0.29813					
23	66->73	0.34153	7.8830	157.28	0.0012	-1.8671	-2.1377
24	58->71	-0.23971	7.9522	155.91	0.0143	7.2356	7.6747

	59->71	0.28172					
	60->71	-0.24258					
	61->71	0.26187					
25	59->71	-0.25863	7.9988	155.00	0.0173	-13.9526	-13.9165
	61->71	0.34147					
	62->71	0.35077					
26	68->74	-0.2315	8.0224	154.55	0.0032	-4.1548	-3.1823
	69->74	0.26096					
	69->75	-0.23282					
27	60->71	0.42332	8.0414	154.18	0.0029	12.9053	14.1368
28	60->71	0.22814	8.0535	153.95	0.0264	14.304	9.2347
	67->74	-0.2298					
	68->74	0.28381					
	69->75	-0.26216					
29	70->76	0.46708	8.0653	153.73	0.0057	3.9035	5.1275
	70->78	-0.27892					
30	58->72	0.35341	8.1057	152.96	0.0068	-12.4439	-11.7037
	59->71	0.3454					
31	70->75	-0.3327	8.2164	150.90	0.0115	-7.9978	-9.304
	70->78	0.40738					
32	57->71	0.31444	8.3524	148.44	0.0124	29.5899	30.7002
	58->71	0.33794					
	59->71	0.23812					
	62->71	0.23196					
33	67->73	0.41163	8.4605	146.54	0.2058	-35.9332	-41.9065
	70->79	0.2595					
34	67->73	0.34679	8.4716	146.35	0.1131	-73.0248	-73.7459
	70->76	0.26524					
	70->79	-0.31661					
35	70->74	0.2376	8.5344	145.28	0.0064	4.2796	5.7103
36	65->73	0.36694	8.5552	144.92	0.0108	11.1779	11.8756

Number of the excited states; Only transitions with contribution over 10.0% were listed; Configuration-interaction coefficient; Excitation energy; Wavelength; Oscillator strength; Rotatory strength in length form (10-40 cgs); Rotatory strength in velocity form (10-40 cgs).
